# TMEM9 promotes lung adenocarcinoma progression via activating the MEK/ERK/STAT3 pathway to induce VEGF expression

**DOI:** 10.1038/s41419-024-06669-8

**Published:** 2024-04-25

**Authors:** Zhiqian Wang, Peng Zhao, Kaihua Tian, Zhongshi Qiao, Hao Dong, Jie Li, Zitong Guan, Hui Su, Yang Song, Xuezhen Ma

**Affiliations:** 1 Department of Oncology, Medical College of Qingdao University, Qingdao, Shandong China; 2 Department of Oncology, Qingdao Central Hospital, University of Health and Rehabilitation Sciences, Affiliated Qingdao Central Hospital of Qingdao University, Qingdao, Shandong China; 3 Biotherapy Center, Qingdao Central Hospital, University of Health and Rehabilitation Sciences, Qingdao, Shandong China; 4 Department of Thoracic Surgery, The Affiliated Hospital of Qingdao University, Qingdao, Shandong China; 5 Department of Oncology, LiaochengPeople’s Hospital, Liaocheng, Shandong China; 6 Department of Nutrition and Food Hygiene, School of Public Health, Medical College of Qingdao University, Qingdao, Shandong China

**Keywords:** Non-small-cell lung cancer, Cancer

## Abstract

Abnormal Transmembrane protein 9 (*TMEM9*) expression has been identified in various human tumors. However, the prognostic potential and mechanistic role of *TMEM9* in lung adenocarcinoma (LUAD) remain unclear. Here, we first found a significant upregulation of *TMEM9* in LUAD tissues, and *TMEM9* expression was positively correlated with microvessel density (MVD), T stage, and clinical stage. Survival analysis demonstrated *TMEM9* was an independent indicator of poor prognosis in LUAD patients. In addition, downregulation of *TMEM9* suppressed tumor growth and metastasis in vitro and in vivo models, and reduced HUVEC proliferation, migration, and tube formation in a cancer cell/HUVEC coculture model. Furthermore, *TMEM9* upregulated *VEGF* expression, and *VEGF*-neutralizing antibodies reversed HUVEC angiogenesis and cancer cell migration ability caused by overexpression of *TMEM9*. In contrast, recombinant *VEGF* (r*VEGF*) abolished the inhibitory effect of *TMEM9*-knockdown LUAD cells on HUVEC angiogenesis and tumor cell migration. Moreover, we showed that *TMEM9* upregulated *VEGF* expression by activating the mitogen-activated protein kinase/extracellular signal-regulated kinase/*STAT3* (*MEK*/*ERK*/*STAT3*) pathway. Together, our study provides mechanistic insights into the role of *TMEM9* in LUAD and highlights the potential of targeting the *TMEM9*/*MEK*/*ERK*/*STAT3*/*VEGF* pathway as a novel therapy for preventing LUAD progression.

## Introduction

Lung cancer is one of the most common cancers globally, with high morbidity and mortality [1]. Non-small cell lung cancer (NSCLC) accounts for 80%–85% of histological types of lung cancers [2]. The most common type of NSCLC is lung adenocarcinoma (LUAD) [3]. Despite significant advances in diagnosing and treating LUAD in recent years, the prognosis of affected patients remains gloomy, particularly in those with recurrent or metastatic LUAD with a poor long-term prognosis [4]. Therefore, it is of great significance to explore the molecular mechanisms leading to an increased metastatic phenotype.

Transmembrane protein 9 (*TMEM9*), a type I transmembrane protein situated in late endosomes and lysosomes, spans 183 amino acids and features a signal peptide and three glycosylation sites at the amino terminus [5]. *TMEM9* has been implicated in various cellular mechanisms, including inflammation [6], tissue regeneration [7], cell differentiation, and tumorigenesis [8]. Many studies have demonstrated that *TMEM9* is highly expressed in multiple human cancers, such as mammary tumors and hepatocellular and colorectal cancer [9]. It is critical in cancer cell proliferation, apoptosis, invasion, metastasis [10], and drug resistance [11]. Therefore, a major function for *TMEM9* in regulating tumorigenesis and development has been suggested. However, little is known about the pathological role of *TMEM9* in LUAD. In addition, the molecular mechanisms of *TMEM9*-elicited tumor development remain unclear.

In the present study, we evaluated *TMEM9* expression and its prognostic value in LUAD. We also provided a novel insight into a mechanism by which *TMEM9* mediated tumor growth via *MEK*/*ERK*/*STAT3*/*VEGF* pathway in LUAD. These findings may represent a novel strategy for therapeutic interventions for LUAD in the future.

## Materials and methods

### Cell culture and transfection

Human umbilical vein endothelial cells (HUVECs), LUAD cell lines A549 and Anip973, and Human lung epithelial cell line BEAS-2B were donated from the Central Laboratory of Affiliated Central Hospital of Qingdao University. A549 was cultured in the RPMI 1640 medium (GIBCO), and Anip973 and BEAS-2B were cultured in the DMEM medium (GIBCO). HUVECs were cultured in HUVEC media (Haixing Biosciences, Jiangsu, China, TCH-G406). All cells were cultured in a 37 °C, 5% CO_2_ incubator supplemented with 10% fetal bovine serum, 100 U/mL of penicillin, and 100 mg/mL of streptomycin.

A549 and Anip973 cells were transfected with short hairpin RNAs (shRNA) targeting *TMEM9* using lentivirus vector GV493 (Genechem, Shanghai, China). The shRNA sequences were as follows: sh*TMEM9*#1, GCATCTGTCCACCTTATAGAA, sh*TMEM9*#2, GACAGTCTTCGATCGGCACAA. The *TMEM9* over-expressing cell lines were generated by lentivirus vector GV703 containing the full-length sequence of *TMEM9* (Genechem, Shanghai, China). For the rescue experiments, an RNAi-resistant *TMEM9*-expression plasmid (oe-TMEM9) or its empty vector (oe-NC) was purchased from Youbio Biological Technology (Hunan, China).

### Clinical specimens

This study collected 43 pairs of fresh LUAD tissues and adjacent normal tissues (ANT) from the Affiliated Hospital of Qingdao University from April to October 2021. Clinicopathological information for these patients is shown in Supplementary Table S1. This study was approved by the Ethics Committee Medical College of Qingdao University (approval number: QDU-HEC-2021145), and informed consent was obtained from each patient. Three LUAD cancer tissue microarrays were purchased from Shanghai Outdo Biotech (Shanghai, China). One tissue microarray (HLugA180Su11) contained 90 pairs of carcinoma tissues and matched para-carcinoma tissues. The other two identical sets of tissue microarray chips (HLugA120PG01) each had 120 LUAD tissue dots, of which 103 could be used as samples for further analysis. The clinical characteristics of these patients are provided in Supplementary Tables S2 and S3.

### Immunohistochemistry (IHC)

Immunohistochemistry was conducted according to the previous protocols [12]. Briefly, the tissue array specimen was first dewaxed, repaired with EDTA, and incubated with primary antibody (anti-*VEGF*, ab1316, 1:200; anti-*CD31*, ab182981, 1:2000 from Abcam; anti-*TMEM9*, A61446, 1:2000 from EpigenTek) overnight at 4 °C. Next, a secondary antibody was added and incubated at room temperature for 60 min. After diluted diaminobenzidine (DAB) was added to pathological sections, hematoxylin (SIGMA) was used for inhibition. Finally, the slices were sealed after dehydration.

The specimens were scored by 3 independent pathologists who did not know any prognosis or clinicopathologic variables. The staining intensity was scored as previously described: 0, no staining; 1, faint cytoplasmic staining; 2, moderate cytoplasmic staining; and 3, strong cytoplasmic staining, and the percentage of stained cancer cells was recorded. The expression level of *TMEM9* and *VEGF* was evaluated by the percentage of positive cells and the staining intensity score. Based on CD31 staining, the microvascular density (MVD) of LUAD tumor sections was assessed according to the previous literature [13].

### In vivo tumorigenesis and metastasis assays

Purchased 4 week-old BALB/c female nude mice were randomly divided into different groups after adaptive feeding for 1 week. A549 cells stably expressing shNC or sh*TMEM9* lentivirus vectors were injected subcutaneously into the left flank of 5-week-old mice (2 × 10^7 ^mL^−1^, 0.1 mL per mice, *n*= 5 in each group). The tumor volume was measured every 4 days and calculated as follows: volume = (width^2^ × length) / 2. 28 days later, the tumor tissue was fixed in 10% formalin solution for 24 h, embedded in paraffin. IHC staining was performed.

To verify the effect of *TMEM9* on tumor metastasis in vivo, A549 cells stably expressing shNC or sh*TMEM9* lentivirus vectors were injected into the tail vein of nude mice (1 × 10^7 ^mL^−1^, 0.15 mL per mice, *n* = 5 in each group). After 6 weeks, lung tissues were dissected, and metastatic nodules were counted. Then it was fixed in 4% paraformaldehyde and analyzed by H&E staining after paraffin embedding. The animal study was approved by the Committee on Qingdao University (approval number: QDU-AEC-2021184).

### Enzyme-linked immunosorbent assay (ELISA)

The cell culture supernatant was collected, and the *VEGF* concentration was determined according to the ELISA kit instructions (Liankebio, Hangzhou, China).

### RNA isolation and quantitative real-time PCR analysis

Total RNA from LUAD cells and tumor tissues was extracted using TRIzol Reagent (Vazyme, Nanjing, China), and cDNA was synthesized by reverse transcription. Finally, quantitative detection was performed with ChamQ Universal SYBR qPCR Master Mix (Vazyme Biotech, Nanjing, China). Relative gene expression was determined by normalizing the expression of each target gene to *β-actin*. The data were analyzed by using 2^△△Ct^.

Primers were shown as follows: *TMEM9*, Forward (F): 5′-GGGCACATTTACAACCAG-3′, Reverse (R): 5′-ATCAGGAAGGCCATG-TAG-3′; *VEGF*, Forward (F): 5′- TTCTGGGCTGTTCTCGCTTC-3′, Reverse (R): 5′- CTCTCCTCTTCCTTCTCTTCTTCC -3′.

### Immunofluorescence

A549 and Anip973 cells were seeded on glass coverslips in 24-well plates. When the cells grew to 80% confluence, they were fixed with 4% paraformaldehyde and permeabilized with 0.1% Triton X-100 in PBS. Fixed cells were incubated with primary *TMEM9* antibody (HPA008483, 1:500 from ATLAS ANTIBODIES) overnight at 4 °C and followed by incubation with FITC-conjugated anti-rabbit IgG for 1 h. DAPI was used for nuclear counterstaining. And cells were observed under a fluorescence microscope.

### CCK8 assay

Cells were seeded in 96-well cell culture plates at 3000 cells per well, and three duplicate wells were set in each group. After 24–72 h of culture, CCK8 (USA, MedChem Express, HY-K0301) reagent was added and cultured for 4 h. The absorbance (OD) at 450 nm was detected with a microplate reader. All experiments were repeated at least 3 times.

### EdU assay

HUVEC proliferation was measured using an EdU assay kit (Beyotime Biotechnology, Shanghai, China). HUVECs were seeded at the appropriate density in a 24-well plate. Subsequently, Transwell chambers containing the transfected LUAD cell were inserted into the 24-well plates and cocultured at 37 °C, 5% CO_2_ for 48 h. EdU staining was performed following the manufacturer’s instructions. Finally, fluorescence images were obtained using a fluorescence microscope.

### Protein extraction and western blotting

LUAD cells were lysed in RIPA buffer containing protease inhibitors and phosphatase inhibitors. Cell lysates were electrophoresed in 12% SDS-PAGE and then transferred onto a nitrocellulose membrane (MerrckMillipore, Ireland). Membranes were blocked with TBST (Tris-buffered saline, 0.1% Tween-20) containing 5% skim milk for 2 h and were incubated with the indicated antibodies, including anti-*MEK1/2* (Abcam, CAT# ab178876);anti-Phospho-*MEK1/2* (CST, CAT# 9154 T);anti-*ERK1/2* (CST, CAT#4695 T); anti-Phospho-*ERK1/2*(CST, CAT#4370 T); anti-*AKT* (CST, CAT#4691 T); anti-Phospho-*AKT* (CST, CAT#4060 T);anti-*STAT3* (Huaan Biotech, ET1607-38); anti-Phospho-*STAT3* (CST, CAT#9145 T) overnight at 4 °C. Then, the membranes were washed with TBST and incubated with a peroxidase-conjugated second antibody for 1 h. The protein bands were detected by enhanced chemiluminescence.

### Chromatin immunoprecipitation (ChIP)

The cells were used to carry out ChIP assays with the SimpleChIP® Enzymatic Chromatin IP Kit (Agarose Beads) (CST, CAT#9002) and an anti-Phospho-*STAT3*(CST, CAT#9145 T) following the provided protocol. The enriched DNA was measured using qRT-PCR with the primers 5′-AAGGCCAGGGGTCACTCCAG-3′ (Forward) and 5′-CCCGCGGGGCATTGGCGAGG-3′ (Reverse) to detect *VEGF* promoter.

### Wound Healing

LUAD cells or HUVECs were seeded in 6-well plates in a CO_2_ incubator at 37 °C. When the cells reached 90–100% confluence, a scratch was made in the monolayer using a sterile p200 tip, and cell debris was washed with PBS. The fresh culture medium containing 2% serum was added to the cells. The cells were cultured for 24–72 h. Images of the scratch were taken at 0 h,24 h, and 72 h using a phase contrast inverted microscope. Then wound healing rates were calculated.

### Transwell coculture system

For transwell chamber coculture, HUVECs were added into each well of a six-plate well, and the transfected LUAD cells were seeded into the upper compartment. In some cases, 150 ng/ml Bevacizumab (Roche Pharmaceuticals, Basel, Switzerland) or 2 ng/ml recombinant human *VEGF*_*165*_ (Peprotech, Suzhou, China) was added into the conditioned medium. These two cells were cocultured for 48 h, and HUVECs were collected for further study.

### Tube formation

HUVECs (6 × 10^5^ cells/ml) were seeded into 96-well plates containing matrix (50uL matrix per well, placed in 37 °C, 5% CO_2_ incubator for 30 min and then used), 50uL cell suspension was added into each well, and 3 repeat wells were set up. The cells were cultured in 37 °C, 5% CO_2_ incubator for 6 h and then photographed under the microscope. The images were evaluated using the Image J software.

### Bioinformatics

The expression level of *TMEM9* in tumor tissues and normal tissues was obtained from the Oncogene Atlas (TCGA) and Genotype-Tissue Expression (GTEx). Kaplan-Meier plotter (www.kmplot.com) [14] was used to examine correlations between *TMEM9* expression and survival. The R software package ggstatsplot realized the correlation chart between *TMEM9* and *VEGF*. RNA-sequencing expression (level 3) profiles for LUAD were downloaded from the TCGA Dataset (https://portal.gdc.com).

### Statistical analysis

All data were obtained from 3 independent experiments. The *t*-test was used to compare the groups. A Cox regression analysis was used to perform univariate and multivariate survival analyses. Data were analyzed using GraphPad Prism 8.0 and IBM SPSS Statistics 25.0 software. NS, no significant difference, *P < 0.05, **P < 0.01.

## Results

### High *TMEM9* mRNA expression is correlated with poor prognosis in LUAD patients

**Fig. 1:**
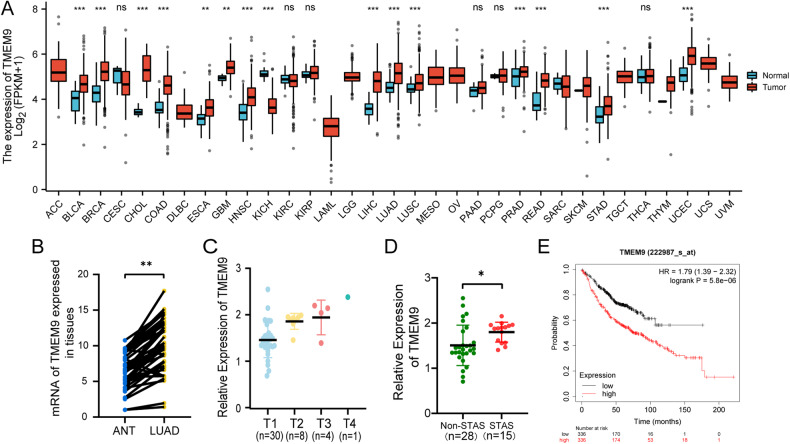
*TMEM9* mRNA expression is upregulated and predicts poor outcomes. **A** *TMEM9* mRNA expression levels in pan‐cancer from TCGA + GTEx database. **B** The mRNA level of *TMEM9* in LUAD and para-carcinoma tissues was measured by qPCR. **C** *TMEM9* mRNA levels in tumor tissues of patients with different T stages. **D** *TMEM9* mRNA levels in tumor tissues of LUAD patients in the STAS negative and STAS positive groups. **E** Kaplan-Meier curves for LUAD patients’ OS depend on *TMEM9* mRNA levels in the TCGA database. Ns, no significant difference, *P < 0.05, **P < 0.01.

To investigate the differences in *TMEM9* expression between tumors and normal tissues, we analyzed the expression levels of *TMEM9* in different tumors and their corresponding normal tissues of pan‐cancer using transcriptomic data from TCGA and GTEx databases. The pan-cancer analysis demonstrated that *TMEM9* was highly expressed in most tumor tissues (Fig. 1A). We also analyzed *TMEM9* mRNA expression in LUAD and pair-matched adjacent normal tissues using the TCGA database and found that *TMEM9* expression was substantially higher in LUAD relative to adjacent normal lung tissues (*P* = 2.2e-05, Supplementary Fig. S1A). We further detected *TMEM9* mRNA expression in 43 LUAD tissues and paired normal lung tissues. The result showed that *TMEM9* was highly expressed in the tumor tissues (*P* < 0.01, Fig. 1B). Subsequently, we evaluated the correlation between *TMEM9* mRNA expression and clinicopathologic parameters in LUAD patients. As shown in Fig. 1C, D, *TMEM9* expression was significantly correlated with the T stage (*P* = 0.029) and spread through air spaces (STAS) (*P* = 0.001) of LUAD (Table S1). The Kaplan-Meier plotter database was then used to evaluate whether *TMEM9* mRNA expression was correlated with the prognosis of LUAD patients. The Data showed that patients with low *TMEM9* expression had longer overall survival (OS) compared to patients with high *TMEM9* expression (*P* = 5.8e-06, Fig. 1E). Therefore, these results suggest that elevated *TMEM9* mRNA expression is a risk factor for LUAD and may be a potential prognostic biomarker for patients with LUAD.

### *TMEM9* was an independent indicator of poor prognosis of LUAD patients

**Fig. 2:**
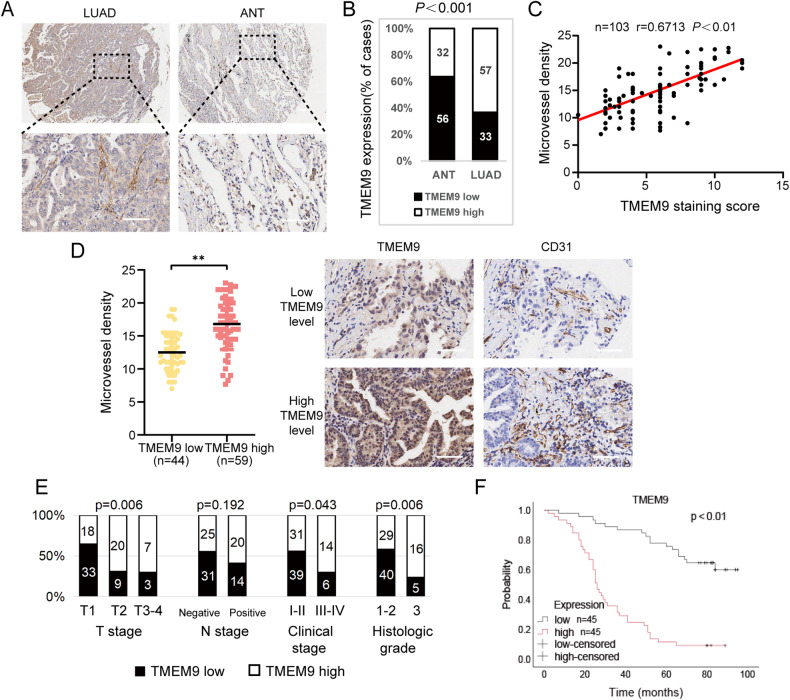
*TMEM9* is overexpressed in tumor tissues and associated with poor prognostic outcomes in LUAD. **A** Representative images of immunohistochemical staining for *TMEM9* expression in LUAD tissues and adjacent normal tissues (ANT). Scale bar, 100 μm. **B** The percentages of high-*TMEM9* cases in LUAD and ANT. **C** *TMEM9* expression levels were positively correlated with MVD levels in tissue samples from patients with LUAD (*n* = 103). **D** The expression of MVD in patients with high *TMEM9* expression was higher than those in patients with low *TMEM9* expression (*n* = 103). Scale bar, 100 μm. **E** The associations between *TMEM9* expression and the clinicopathological parameters of LUAD patients were analyzed. **F** Kaplan-Meier curves of *TMEM9* expression in LUAD (*n* = 90). Data are presented as the mean ± SD. Ns, no significant difference, *P < 0.05, **P < 0.01.

The immunohistochemistry for *TMEM9* was performed in the LUAD specimens chip. The staining intensity was higher in LUAD compared with peritumor tissues (*P* < 0.01, Fig. 2A, B). As shown in Fig. 2C, D, high *TMEM9* expression was significantly associated with high microvessel density (MVD) in LUAD tissues (*P* < 0.01). In addition, the expression of *TMEM9* increased gradually with the progress of the clinical stage, T stage, and grade (Fig. 2E and Supplementary Fig. S1B, C). Kaplan-Meier curves showed that LUAD patients with high *TMEM9* expression had shorter overall survival (*P* < 0.01, Fig. 2E). Furthermore, univariate and multivariate Cox regression analysis showed that *TMEM9* expression was an independent prognostic factor for overall survival of LUAD patients (Table 1). Together, these results suggest that increased *TMEM9* expression is a poor prognostic factor for LUAD.

**Table 1:** Univariate and multivariate analysis of different prognostic factors for overall survival in patients with LUAD.

Characteristics	Total patients(*n*,%)	Univariate analysis		Multivariate analysis	
		OS HR (95% CI)	*P*-value	OS HR (95% CI)	*P*-value
**Gender**					
Male	48 (53.3%)	Reference			
Female	42 (46.7%)	0.885(0.528–1.484)	0.644		
**Age,year**					
<60	40 (44.4%)	Reference			
≥60	50 (55.6%)	1.476(0.868–2.510)	0.150		
**T stage**					
T1-2	80 (88.9%)	Reference			
T3-4	10 (11.1%)	3.473(1.666–7.238)	**0.004**	3.246 (1.329–7.928)	**0.010**
**N stage**					
Negative	56 (62.2%)	Reference			
Positive	34 (37.8%)	1.558(0.917–2.645)	0.101		
**Clinical stage**					
I -II	70 (77.8%)	Reference			
III-IV	20 (22.2%)	2.482 (1.382–4.460)	**0.002**	1.251 (0.593–2.639)	0.556
**Histologic grades**					
1–2	69 (76.7%)	Reference			
3	21 (23.3%)	2.064(1.164–3.659)	**0.013**	1.282 (0.673–2.444)	0.450
***TMEM9***					
Low	45 (50%)	Reference			
High	45 (50%)	2.286(1.340–3.901)	**0.002**	5.925 (3.212–10.929)	**0.001**

*TMEM9*, transmembrane protein 9; LUAD lung adenocarcinoma.

### *TMEM9* promotes LUAD cell growth, migration, and angiogenesis

**Fig. 3:**
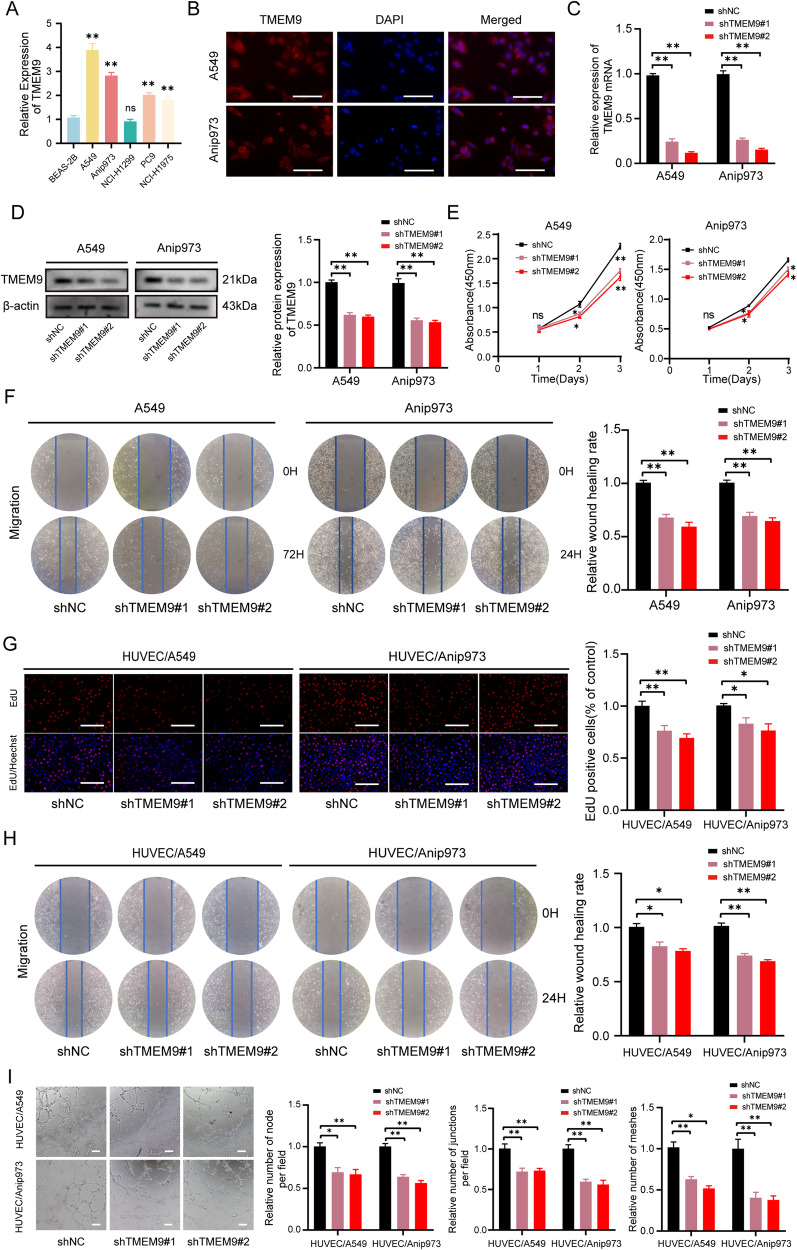
*TMEM9* knockdown decreased LUAD cell growth, migration, and angiogenesis. **A** The mRNA levels of *TMEM9* in LUAD and normal cells were measured by qPCR. **B** Immunofluorescent staining showed subcellular localization of *TMEM9* in A549 and Anip973. Scale bar, 100 μm. **C** QPCR analysis of *TMEM9* in LUAD stable cell lines with *TMEM9* knockdown (sh*TMEM9*) or their control cell lines (sh-NC). **D** Western blot analysis of *TMEM9* in LUAD stable cell lines with *TMEM9* knockdown (shTMEM9) or their control cell lines (sh-NC). **E** The CCK8 assays showed that *TMEM9* knockdown significantly inhibited LUAD cell proliferation. **F** Scratch wound healing assay showed that *TMEM9* knockdown inhibited the migration of LUAD cells. **G**–**I** ShNC cells or sh*TMEM9* cells were cocultured with HUVECs for 48 h, and changes in angiogenesis were evaluated by EdU (**G**), wound-healing (**H**), and tube formation (**I**) assays. Scale bar, 100 μm. Results are shown for three experiments performed in triplicate.Data are presented as the mean ± SD. Ns, no significant difference, *P < 0.05, **P < 0.01.

*TMEM9* expression was examined in five different LUAD cell lines as well as normal lung epithelial cells BEAS-2B. The expression level of *TMEM9* in LUAD cell lines was significantly increased compared with normal lung epithelial cells (Fig. 3A). Immunofluorescent staining showed that *TMEM9* predominantly localized to the cytoplasm and the perinucleus with a speckled pattern in A549 and Anip973 cells (Fig. 3B). Next, we determined the biological function of *TMEM9* in LUAD cells. *TMEM9* was knocked down in A549 and Anip973 (Fig. 3C, D). As shown in Fig. 3E, F, CCK8 and wound-healing assay indicated that down-regulation of *TMEM9* inhibited the proliferation and migration of LUAD cells.

To explore the effects of *TMEM9* on the Matrigel capillary structure formation of HUVECs, an indirect coculture model was established based on a transwell chamber. *TMEM9* knockdown LUAD cells were cocultured with HUVECs, and then the proliferation, migration, and tube formation of HUVECs were determined. These results showed that *TMEM9* interfering in LUAD cells significantly decreased the proliferation, migration, and tube formation of HUVECs compared to scramble shRNA interfering (Fig. 3G–I).

**Fig. 4:**
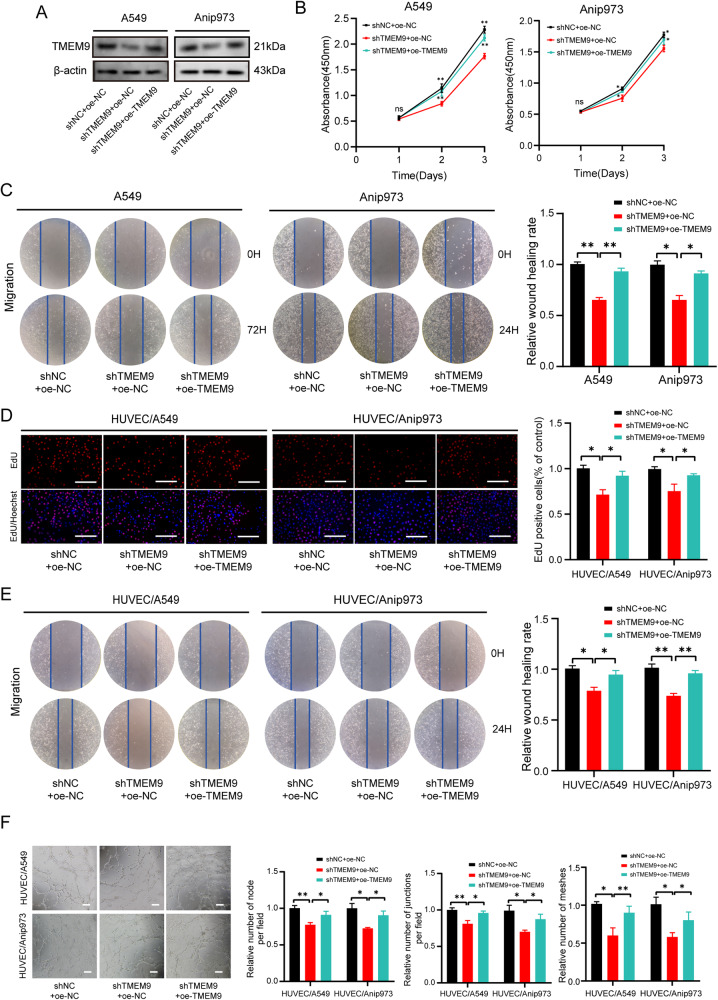
Upregulated *TMEM9* expression could partly reverse the inhibitory effects of *TMEM9* knockdown on cell growth, migration, and angiogenesis. **A** *TMEM9* stable knockdown LUAD cells were transfected with a vector expressing *TMEM9*.The expression of *TMEM9* in A549 and Anip973 cells after transfection was detected by Westen blot. **B** The CCK8 assays showed that upregulated *TMEM9* expression could reverse the inhibitory effects of *TMEM9* knockdown on cell proliferation. **C** Scratch wound healing assay showed that upregulated *TMEM9* expression could reverse the inhibitory effects of *TMEM9* knockdown on cell migration**. D**–**F** LUAD cells were cocultured with HUVECs for 48 h, and the effect of rescued *TMEM9* expression in LUAD cells on angiogenesis were evaluated by EdU (**D**), wound-healing (**E**), and tube formation (**F**) assays. Scale bar, 100 μm. Results are shown for three experiments performed in triplicate.Data are presented as the mean ± SD. Ns, no significant difference, *P < 0.05, **P < 0.01.

Next, Rescue experiments were performed to further eliminate the possibility that the decrease in proliferation, migration, and angiogenesis was due to shRNA‐mediated off-target effects. Sh*TMEM9*#2 was chosen for the following experiment due to its higher interference efficiency. *TMEM9* stable knockdown cells were transfected with a vector expressing *TMEM9* (Fig. 4A). As shown in Fig. 4B–F, the rescue of *TMEM9* expression could partly reverse the inhibitory effects of *TMEM9* knockdown on cell growth, migration, and angiogenesis. In addition, we established LUAD cells stably overexpressing *TMEM9* (Supplementary Fig. S2A). *TMEM9* overexpression promotes LUAD cell growth, migration, and angiogenesis (Supplementary Fig. S2B–F). These results indicate that *TMEM9* may serve an important role in LUAD progression.

### *TMEM9* increases the migration of LUAD cells and promotes angiogenesis through *VEGF* secretion

**Fig. 5:**
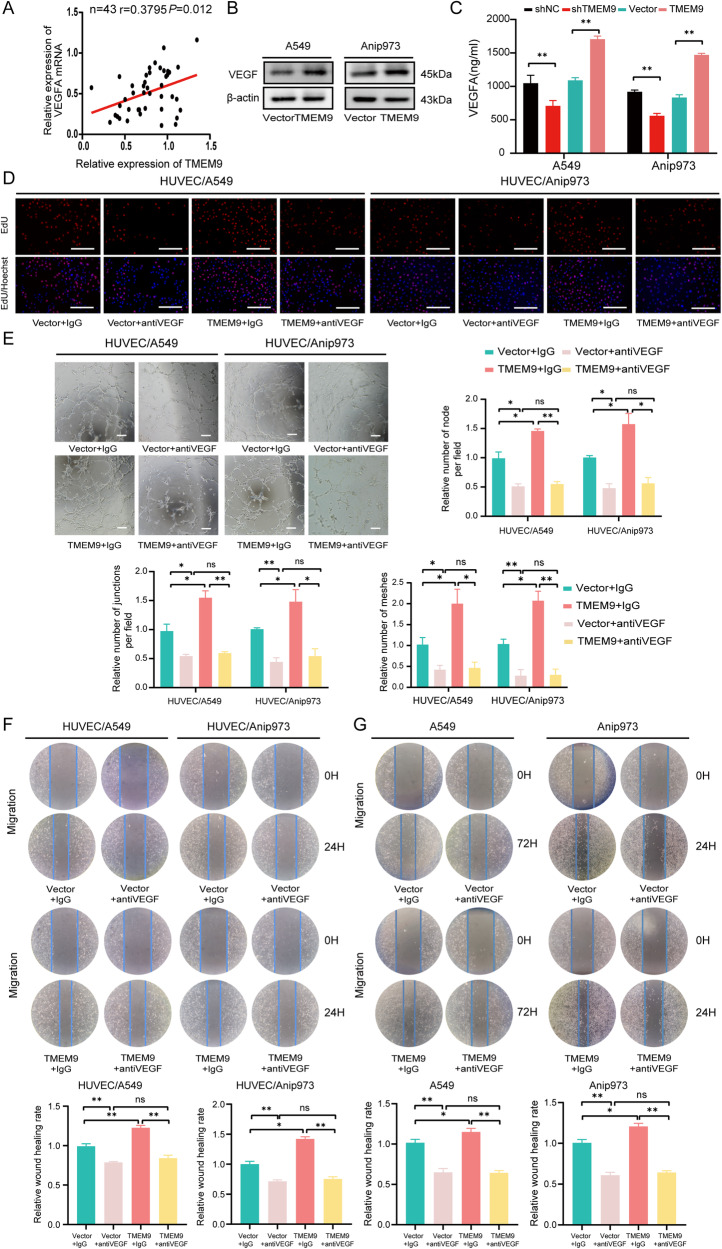
*TMEM9* increases the migration of LUAD cells and promotes angiogenesis through *VEGF* secretion. **A** A significant association of *TMEM9* expression with *VEGF* in LUAD tissue was observed (*n* = 43). **B** *TMEM9* overexpressing increased *VEGF* expression in LUAD cells. **C** Culture supernatant *VEGF* levels were determined by ELISA. **D**–**F** The EdU assay (**D**), tube formation (**E**), and scratch surface healing (**F**) were examined. anti*VEGF* was added to the culture medium of HUVECs coculture with *TMEM9* overexpressed LUAD cells or control cells. **G** Effect of Bevacizumab (Bev) on LUAD cell migration. Results are shown for three experiments performed in triplicate. Data are presented as the mean ± SD. Ns, no significant difference, *P < 0.05, **P < 0.01.

*VEGF* is one of the critical regulators responsible for angiogenesis [15]. To test the hypothesis that *VEGF*, as a downstream of *TMEM9*, might enhance the angiogenesis, we first analyzed the association of *TMEM9* with *VEGF* using public databases. The data showed a positive correlation between *VEGF* expression level and *TMEM9* level in LUAD (*P* = 0.004, Supplementary Fig. S3). We further detected the mRNA expression of *TMEM9* and *VEGF* in LUAD tissues by qPCR. As expected, a significant correlation between *TMEM9* and *VEGF* was observed (*P* = 0.012, *r* = 0.3795, Fig. 5A). Overexpression of *TMEM9* significantly increased the expression of *VEGF* in A549 and Anip973 cells (Fig. 5B and Supplementary Fig. S4A). At the same time, the knockdown of *TMEM9* decreased *VEGF* expression (Supplementary Fig. S4B, C). Subsequently, *VEGF* levels in the cell supernatant were detected by ELISA. The results showed that inhibition of *TMEM9* significantly reduced the secretion of *VEGF*, while overexpression of *TMEM9* upregulated *VEGF* (Fig. 5C).

To verify whether *TMEM9* expression in LUAD cell lines promoted tumor cell migration and angiogenesis through *VEGF*, *VEGF* neutralizing antibody (Bevacizumab) was added into the coculture system. The results showed that Bevacizumab reversed the promoting effect of *TMEM9*-overexpressed LUAD cells on HUVEC angiogenesis (Fig. 5D–F and Supplementary Fig. S5B). Moreover, Bevacizumab blocked the positive effect of *TMEM9* on the migration of LUAD cell lines(Fig. 5G). Furthermore, when compared to control, recombinant *VEGF* (r*VEGF*) abolished the inhibitory effect of *TMEM9*-knockdown LUAD cells on HUVEC angiogenesis and tumor cell migration (Supplementary Fig. S5). Together, these results indicate that *VEGF* is a downstream molecule of *TMEM9*, which plays an essential role in migration and angiogenesis.

### *TMEM9* promotes *VEGF* expression via *MEK*/*ERK*/*STAT3* signaling pathway in LUAD

**Fig. 6:**
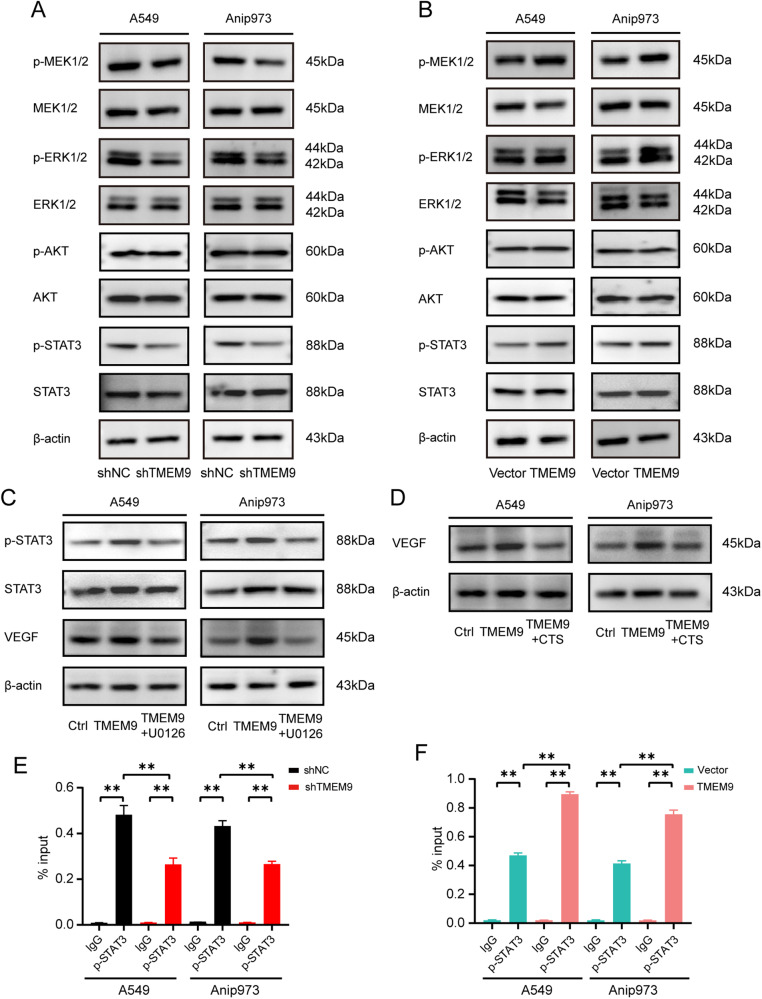
*TMEM9* promotes *VEGF* expression via the *MEK*/*ERK*/*STAT3* signaling pathway in LUAD. **A** The expression of *p-MEK1/2*, *MEK1/2*, *p-ERK1/2*, *ERK1/2*, *p-AKT*, *AKT*, *p-STAT3*, and *STAT3* in LUAD stable cell lines with *TMEM9* inhibition (sh*TMEM9*) or their control cell lines (sh-NC) was analyzed by Western blot. **B** Western blot analysis of *p-MEK1/2*, *MEK1/2*, *p-ERK1/2*, *ERK1/2*, *p-AKT*, *AKT*, *p-STAT3*, and *STAT3* in LUAD stable cell lines overexpressing *TMEM9* (*TMEM9*) or their control cell lines (vector). **C** The protein expression of *p-STAT3*, *STAT3*, and *VEGF* in *TMEM9* overexpressing LUAD cells treated with U0126 was detected by Western blot. **D** The protein expression of *VEGF* in *TMEM9* overexpressing LUAD cells after treatment with cryptotanshinone (CTS) was detected by Western blot. **E**-**F** ChIP assays were performed in *TMEM9* knockdown (**E**) and overexpression (**F**) cells with an anti-*p*-*STAT3* antibody or IgG. Precipitated DNAs were measured by qRT-PCR for *VEGF* promoter.

Previous studies suggested that *VEGF* expression is regulated by several signaling pathways, such as the transcription factor *STAT3*, *MEK*/*ERK*, or *AKT* pathways [16–20]. To elucidate the mechanism of *TMEM9* induced angiogenesis, the activation of *STAT3*, *AKT*, and *MEK/ERK* signaling was observed in LUAD cells treated with sh*TMEM9* LUAD cells. Our results revealed that *TMEM9* knockdown reduced the phosphorylation levels of *MEK1/2, ERK1/2*, and *STAT3* in both A549 and Anip973 cells (Fig. 6A). Conversely, *TMEM9* overexpression increased the levels of phosphorylated *MEK1/2, ERK1/2* and *STAT3* (Fig. 6B). Next, we investigated the effect of *MEK* inhibitor (U0126) and *STAT3* inhibitor (cryptotanshinone, CTS) on *VEGF* expression in LUAD cells mediated by *TEME9*. As shown in Fig. 6C, D, *MEK* inhibitor (U0126) inhibited the promoting effect of *TMEM9* on *STAT3* phosphorylation and *VEGF* expression in LUAD cells. In addition, *STAT3* inhibitor (CTS) inhibited *VEGF* expression mediated by *TMEM9*. Furthermore, we determined the effect of *TMEM9* on *VEGF* expression by *p-STAT3*. ChIP-qPCR showed that the knockdown of *TMEM9* reduced the binding of *p-STAT3* to the *VEGF* promoter (Fig. 6E). Consistently, overexpression of *TMEM9* produced the opposite results (Fig. 6F). Thus, these results suggest that TMEM9 partially promotes *VEGF* expression through the *MEK/ERK/STAT3* pathway in LUAD.

### *TMEM9* knockdown inhibits tumor progression in a xenograft model and a lung metastasis model

**Fig. 7:**
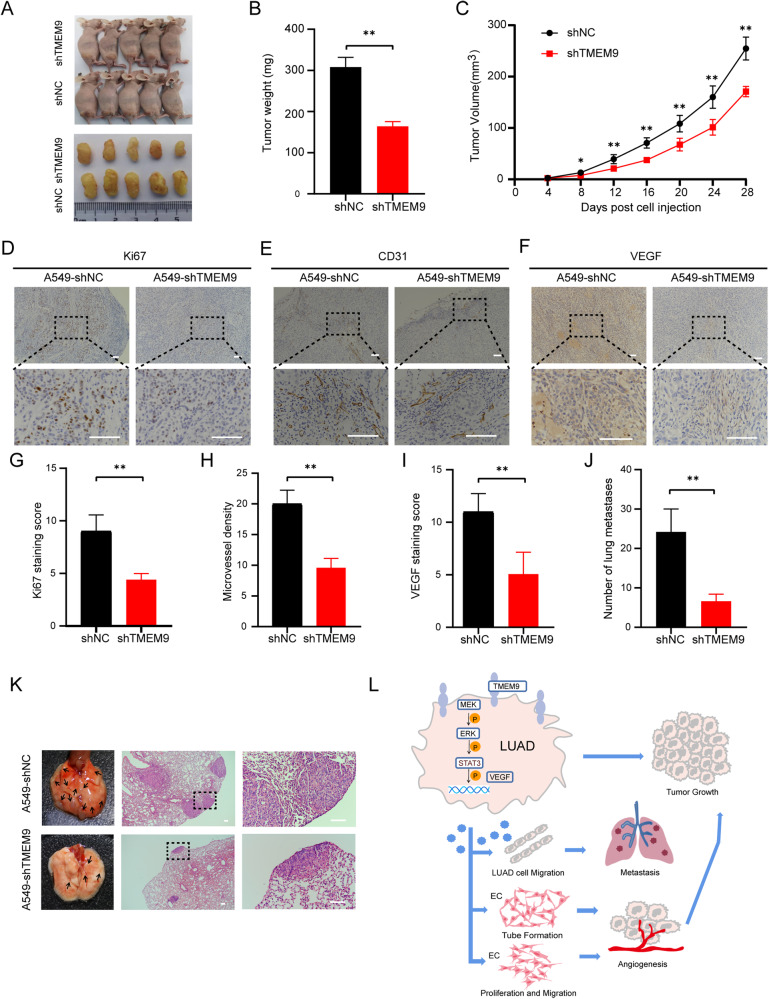
*TMEM9* knockdown inhibits tumor progression in the xenograft and lung metastasis models. **A**–**C** *TMEM9* knockdown A549 cells and control cells were injected subcutaneously into nude mice. Tumor sizes (**A**), weight (**B**), and growth (**C**) were shown. **D-I** A lower expression of *Ki67*, *CD31*, and *VEGF* was detected in the xenograft with *TMEM9* knockdown. **J**-**K** LUAD cells were injected into the tail vein of nude mice. *TMEM9* knockdown significantly reduced the number of pulmonary metastatic foci. **L** Schematic diagram of this study. Each bar represents the mean ± SD. *P < 0.05, **P < 0.01.

To further test the role of *TMEM9* in vivo, *TMEM9* knockdown A549 and control cells were subcutaneously injected into nude mice, respectively. The mice were sacrificed 28 days after injection. As shown in Fig. 7A–C, *TMEM9* knockdown significantly reduced tumor volume and weight compared with the control group. Immunostaining for A549 cells in xenografts revealed that the expression of *Ki67* was decreased markedly in the *TMEM9* knockdown group (Fig. 7D, G). Next, we investigated the association of angiogenesis with *TMEM9* in vivo. IHC staining showed that the knockdown of *TMEM9* also led to a reduced expression of *VEGF* and MVD compared with the control (Fig. 7E–F, H–I).

In addition, we explored the effect of *TMEM9* on lung metastasis. LUAD cells were injected into the tail vein of nude mice, and lung metastases were estimated. We found that *TMEM9* knockdown resulted in fewer metastatic lesions than the control group (Fig. 7J–K). However, there were no significant differences in the expression of *CD31* and *VEGF* in lung metastases between the two groups (Supplementary Fig. S6).

## Discussion

*TMEM9*, a new member of the transmembrane protein family, is involved in inflammation, tissue regeneration, cell differentiation, and proliferation. The role of *TMEM9* in tumors has garnered increasing attention in recent years. Zhang et al. found that high expression of *TMEM9* in HCC patients had a shorter survival period. Downregulation of *TMEM9* in HCC cell lines can inhibit cell growth and metastasis and promote apoptosis, which may be a potential target for HCC therapy [10]. In addition, several studies showed that *TMEM9* could facilitate the assembly of v‐ATPase, which leads to vesicular acidification and lysosomal dysfunction that promotes tumor occurrence in hepatocellular carcinoma and colorectal cancer [7, 9]. Zhang et al. have recently established the significance of the lysosomal *TMEM9*-V-ATPase - regulator - Rag axis as a crucial regulator of *mTOR* signal integrity and proliferation of breast cancer cells [8]. This axis also influences the sensitivity of breast cancer cells to *mTOR* inhibitors, thereby presenting itself as a promising molecular target for breast cancer treatment and a therapeutic target for mTOR inhibitor-resistant patients. Another study reported that *FOXD2-AS1* regulates *TMEM9* to mediate sorafenib resistance in HCC cells [11]. The findings indicate that *TMEM9* may have crucial implications in the processes of tumorigenesis and development. But the role of *TMEM9* in LUAD remains to be investigated. In the study, we investigated the expression and function of *TMEM9* in LUAD and demonstrated that *TMEM9* promoted tumor cell proliferation, migration, and angiogenesis via increasing *VEGF* expression and secretion. Recently, several studies have reported that *TMEM9* was overexpressed in multiple cancers and significantly correlated with poor prognosis [8–10]. Our results showed that *TMEM9* mRNA and protein expression in LUAD tissues was higher than those in normal peritumorial tissues. The expression of *TMEM9* was significantly correlated with clinicopathologic characteristics, including higher T stage, TNM stage, histological grade, and STAS. In addition, we found that *TMEM9* expression was an independent indicator of poor prognosis of LUAD patients, suggesting *TMEM9* was involved in tumorigenesis and progression of LUAD.

It has been reported that *TMEM9* promotes the proliferation of breast cancer and HCC cells [7, 8]. In the study, we demonstrated that *TMEM9* increased the proliferation and migration of LUAD. Angiogenesis has been considered a characteristic feature of rapidly growing solid tumors [21] and is closely associated with tumor growth and metastasis [22, 23]. We first observed a positive correlation between the expression level of *TMEM9* and MVD in patient tumor tissues. In vitro experiments further demonstrated that *TMEM9* knockdown in LUAD cells reduced the proliferation, migration, and tube formation ability of HUVEC. In an animal model, we also found that *TMEM9* knockdown significantly reduced *CD31* expression in the xenograft. These data suggest that *TMEM9* may play an essential role in promoting angiogenesis.

Tumor angiogenesis is a complex process in which multiple cells and cytokines are involved [24]. The interactions between tumor cells and endothelial cells (ECs) influence tumor angiogenesis [25].*VEGF* is one of the most critical regulatory factors of tumor angiogenesis and promotes tumor progression, making it a key target of anticancer therapy in various malignant tumors [26–28]. We discovered that *TMEM9* knockdown inhibited the expression and secretion of *VEGF* in LUAD cells. Bevacizumab reversed the positive effect of *TMEM9* overexpressed LUAD cells on HUVEC angiogenesis, while recombinant *VEGF* abolished the inhibitory effects of sh*TMEM9* LUAD cells on HUVEC angiogenesis. These findings indicate that *TMEM9* promotes LUAD angiogenesis in a *VEGF* dependent manner.

Furthermore, *VEGF* driven from tumor cells also enhances its ability to invade and migrate [29–33]. The present study revealed that the effect of *TMEM9* on the migration of LUAD cells was partially dependent on *VEGF*. Previous studies suggested that *VEGF* expression is regulated by several signaling pathways, such as the transcription factor *STAT3*, *MEK*/*ERK*, or *AKT* pathways [34–36]. Our results showed that *TMEM9* increased the phosphorylation levels of *MEK*, *ERK*, and *STAT3*. Previous studies have shown that *STAT3* is a downstream target of the *ERK* signaling pathway in various tumors [37–40]. Consistent with previous findings, we also found that U0126 treatment reduced protein levels of *p-STAT3* in *TMEM9*-overexpressing LUAD cells. Studies have confirmed that *STAT3* is a direct transcriptional activator of *VEGF* gene [41]. In this study, we demonstrated that *MEK* inhibitor (U0126) and *STAT3* inhibitor (CTS) inhibited the promoting effect of *TMEM9* on *VEGF* expression in LUAD cells. In addition, we confirmed that *TMEM9* can promote *p-STAT3* binding to *VEGF* promoters. Taken together, our data established *TMEM9* as a candidate biomarker for the prognosis of lung adenocarcinoma and favored tumor progression. Targeting *TMEM9*/*MEK*/*ERK*/*STAT3*/*VEGF* pathway may exert some antitumor effect.

### Supplementary information

supplementary materials

Supplementary Figure S1

Supplementary Figure S2

Supplementary Figure S3

Supplementary Figure S4

Supplementary Figure S5

Supplementary Figure S6

## Data Availability

The raw data acquired for this study are available from the corresponding author upon reasonable request.
