# Supplementary material for: TMEM9 promotes lung adenocarcinoma progression via activating the MEK/ERK/STAT3 pathway to induce VEGF expression
Source: Cell Death Dis. 2024 Apr 25;15(4):295. doi: 10.1038/s41419-024-06669-8 (PMC11045738; doi:10.1038/s41419-024-06669-8)
Supplement: Supplementary file 1 — supplementary materials [file 41419_2024_6669_MOESM1_ESM.pdf]

Figure 3D

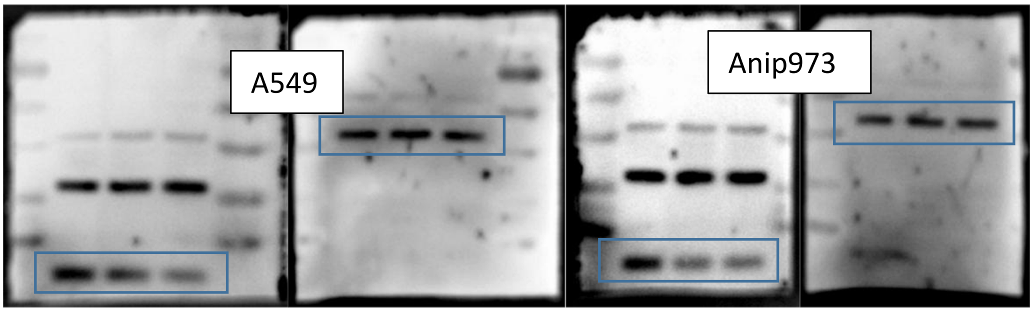

TMEM9 β-actin TMEM9 β-actin

Figure 4A

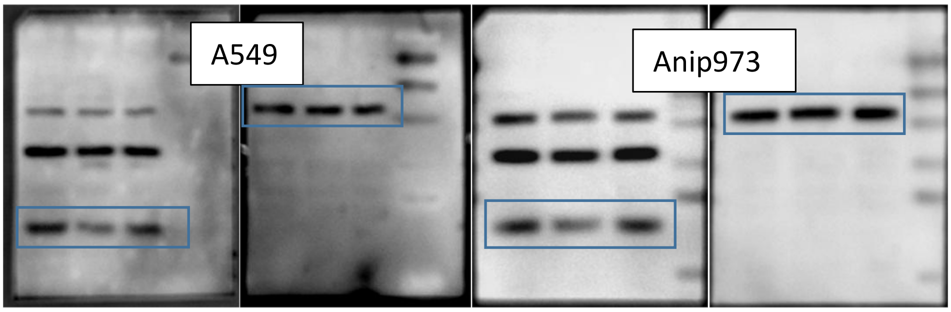

TMEM9 β-actin TMEM9 β-actin

Figure 5B

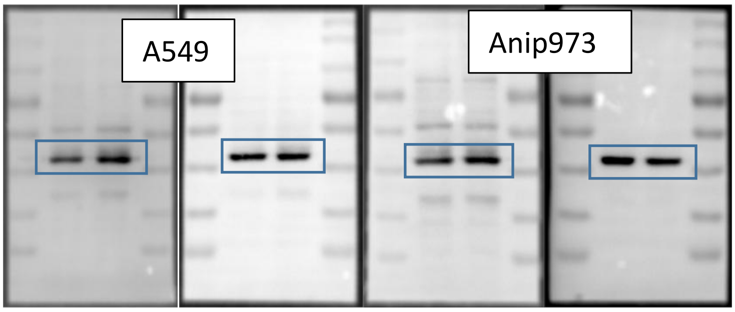

VEGF β-actin VEGF β-actin

Figure 6A

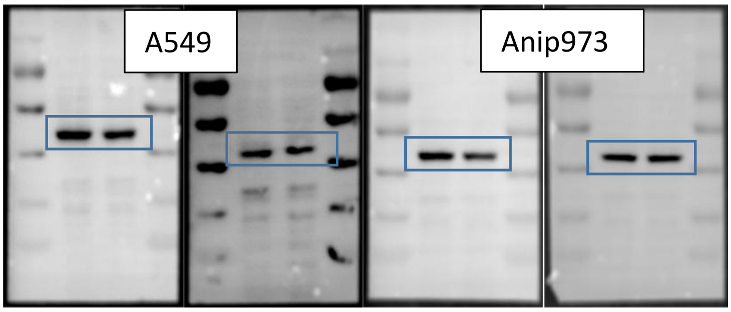

p-MEK1/2 β-actin p-MEK1/2 β-actin

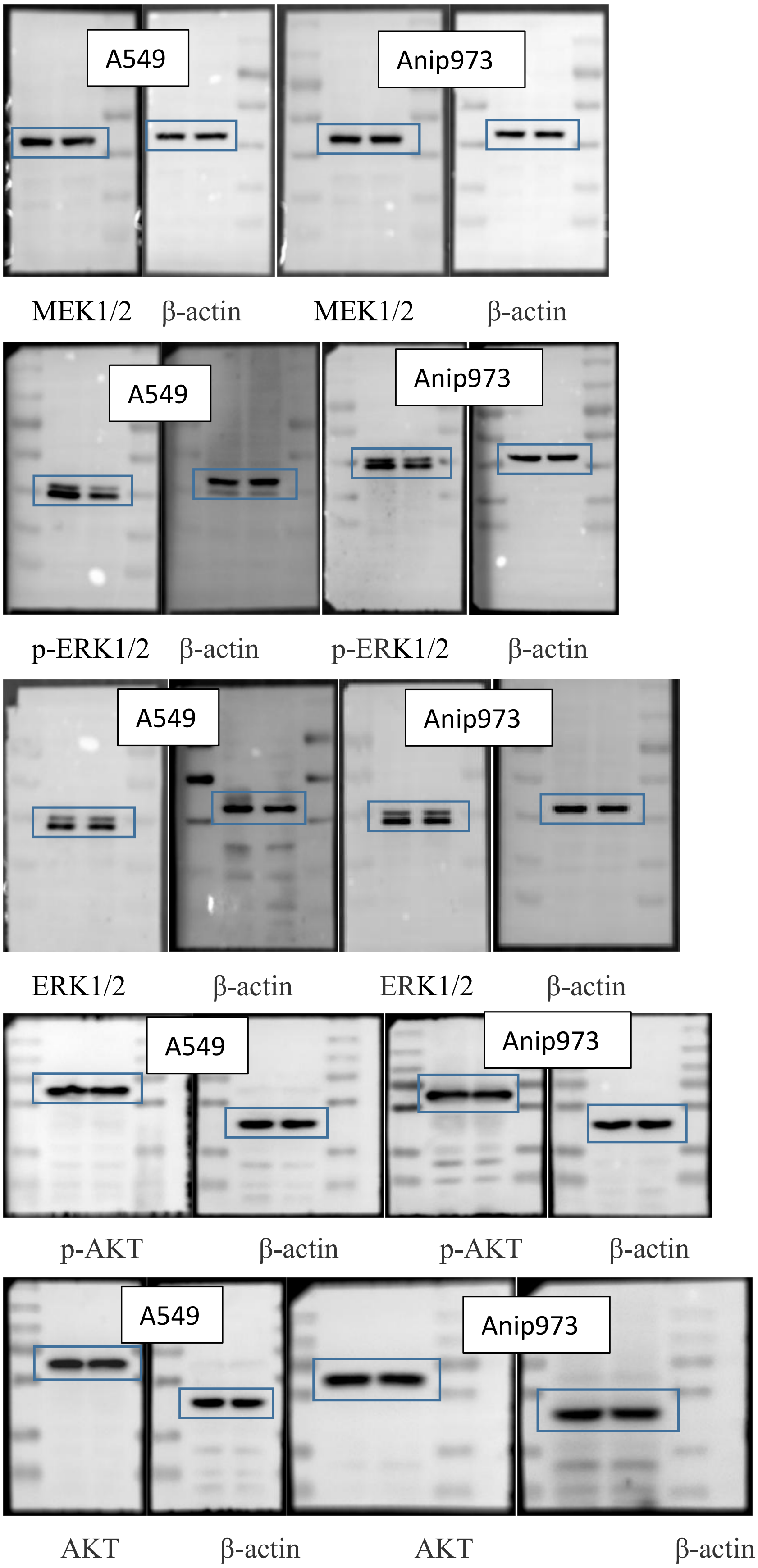
A549
Anip973
MEK1/2
β-actin
MEK1/2
β-actin
A549
Anip973
p-ERK1/2
β-actin
p-ERK1/2
β-actin
A549
Anip973
ERK1/2
β-actin
ERK1/2
β-actin
A549
Anip973
p-AKT
β-actin
p-AKT
β-actin
A549
Anip973
AKT
β-actin
AKT
β-actin

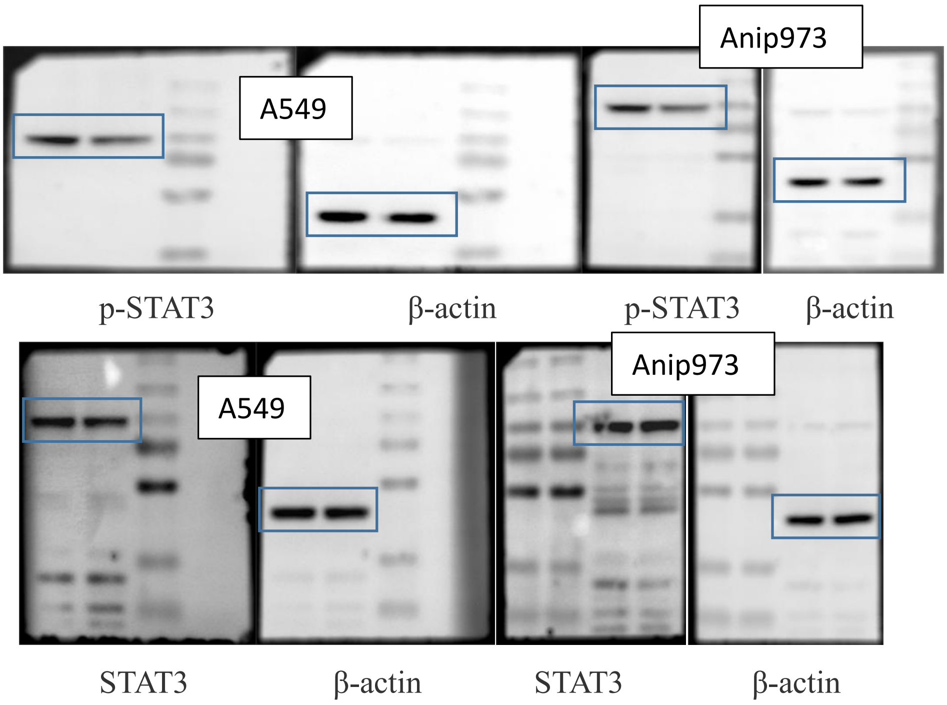
A549
Anip973
p-STAT3
β-actin
p-STAT3
β-actin
A549
Anip973
STAT3
β-actin
STAT3
β-actin

Figure 6B

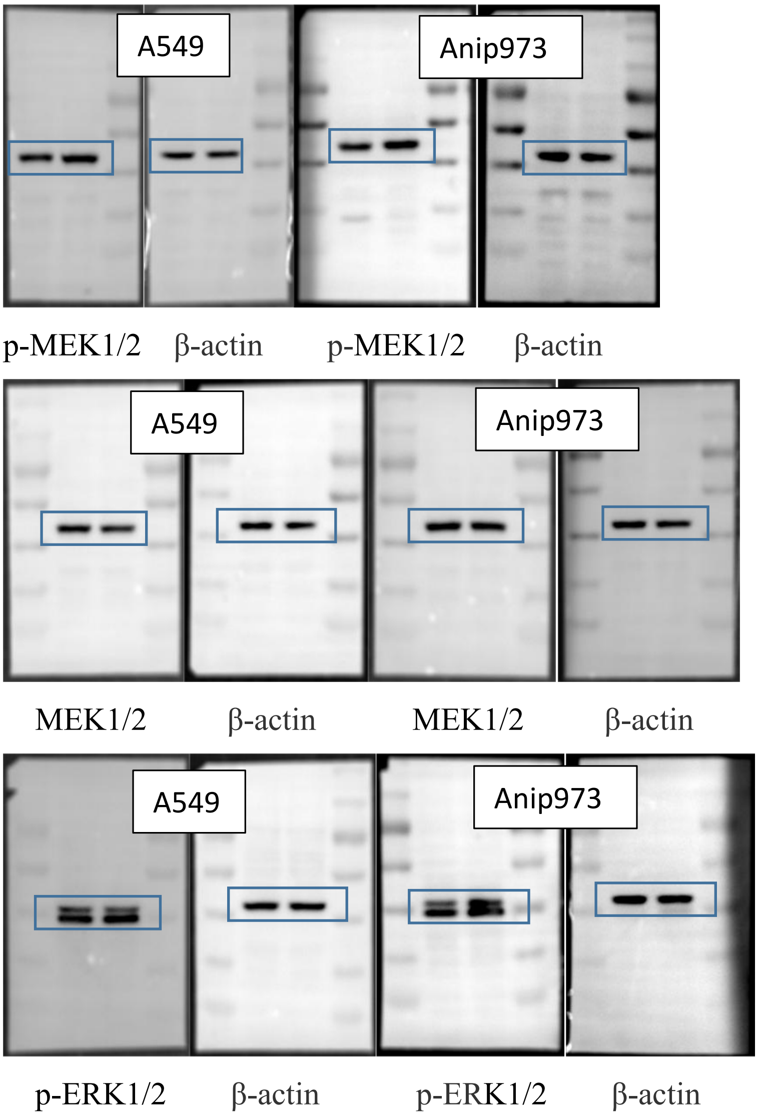
A549
Anip973
p-MEK1/2
β-actin
p-MEK1/2
β-actin
A549
Anip973
MEK1/2
β-actin
MEK1/2
β-actin
A549
Anip973
p-ERK1/2
β-actin
p-ERK1/2
β-actin

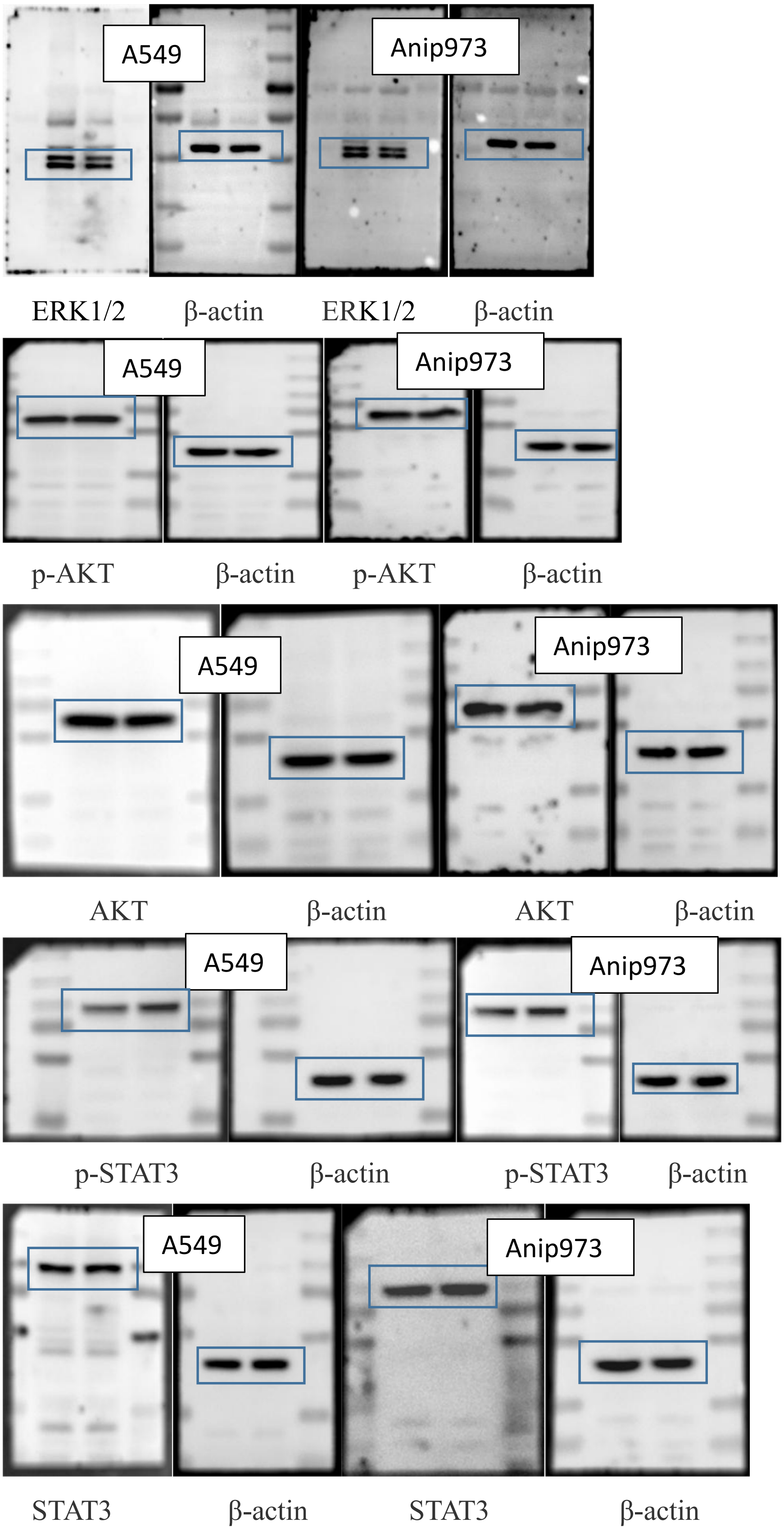
A549
Anip973
ERK1/2
β-actin
ERK1/2
β-actin
A549
Anip973
p-AKT
β-actin
p-AKT
β-actin
A549
Anip973
AKT
β-actin
AKT
β-actin
A549
Anip973
p-STAT3
β-actin
p-STAT3
β-actin
A549
Anip973
STAT3
β-actin
STAT3
β-actin

Figure 6C

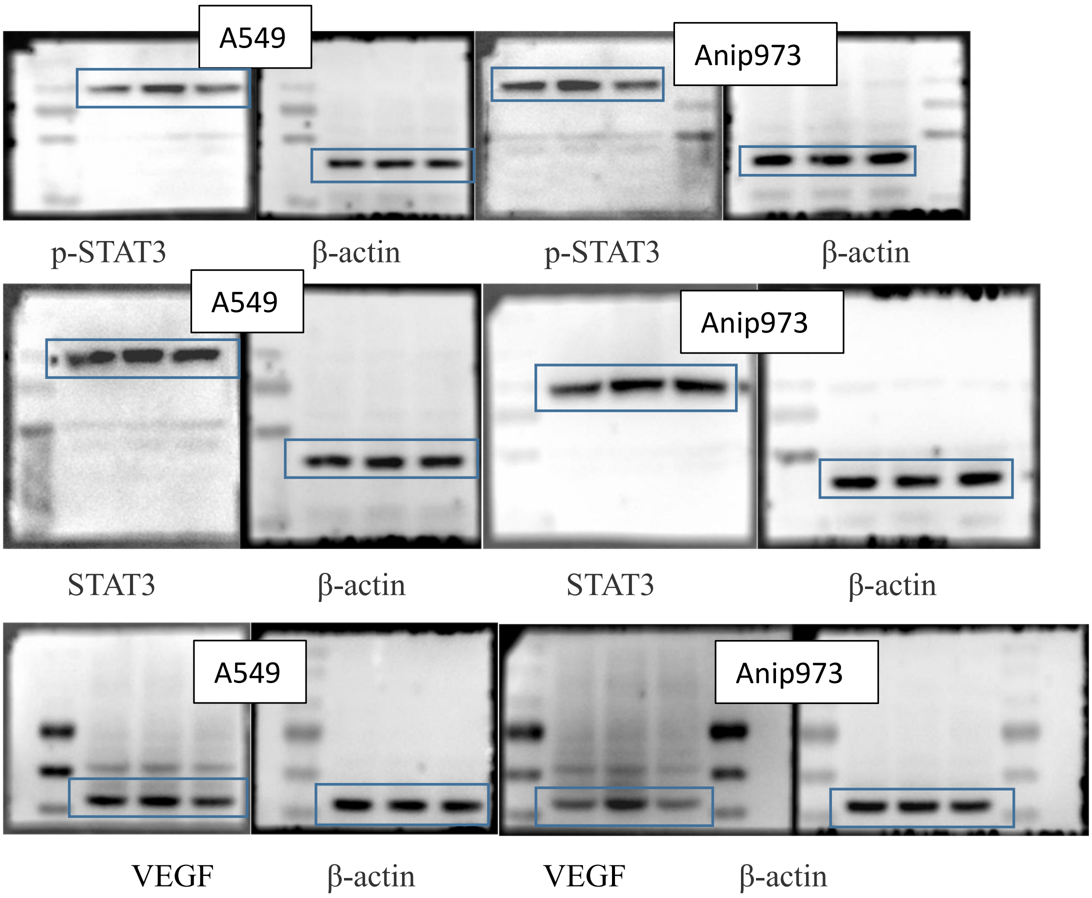

Figure 6D

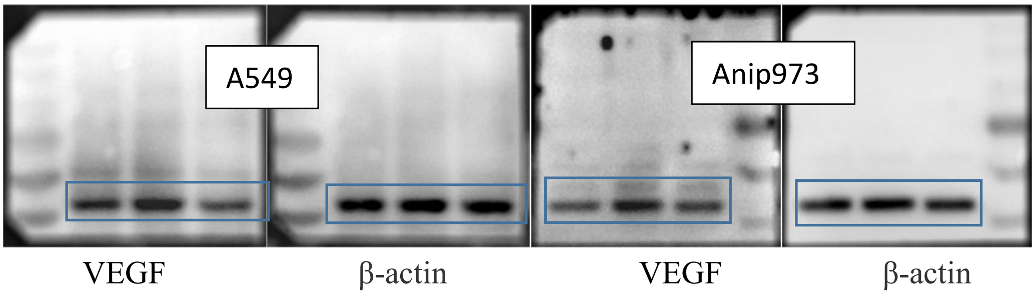

Supplementary Figure S2 A

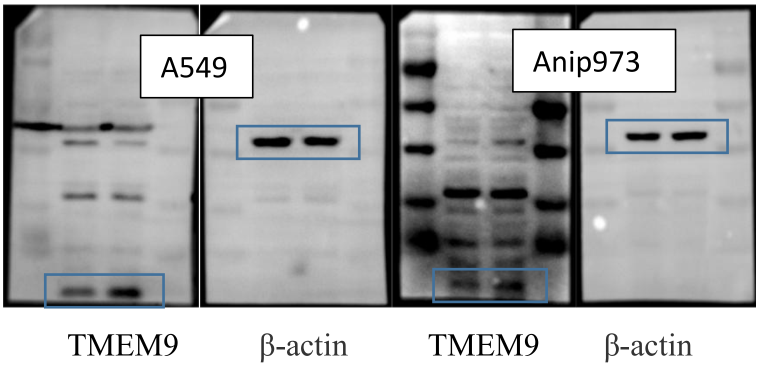

Supplementary Figure S4B

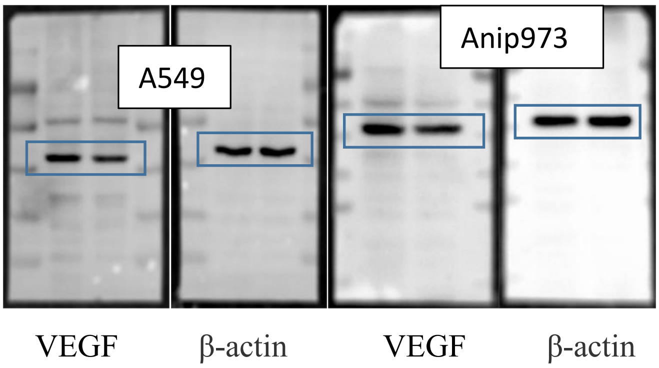
A549
Anip973
VEGF
β-actin
VEGF
β-actin

Supplementary Figure legends

Supplementary Figure S1. *TMEM9* expression is upregulated in clinical LUAD samples. A The expression level of *TMEM9* in paired cancer and normal tissues was obtained from the TCGA database. **B** Representative images of IHC staining for *TMEM9* in different histologic grades of LUAD tissues. Scale bars: 100 μm. **C** Images of IHC staining for *TMEM9* in different T stages of LUAD tissues. Scale bars: 100 μm. ***P*<0.01.

Supplementary Figure S2. *TMEM9* overexpression promoted LUAD cell growth, migration, and angiogenesis. A *TMEM9* expression in A549 and Anip973 was determined by western blot. **B** The CCK8 assays showed that *TMEM9* overexpression significantly enhanced LUAD cell proliferation. **C** Scratch wound healing assay showed that *TMEM9* overexpression enhanced the migration of LUAD cells. **D-F** LUAD stable cell lines overexpressing *TMEM9* (*TMEM9*) or their control cell lines (Vector) were cocultured with HUVECs for 48 hours, and changes in angiogenesis were evaluated by EdU (D), wound-healing (E), and tube formation (F) assays. Scale bar, 100 μm. Results are shown for three experiments performed in triplicate.Data are presented as the mean ± SD. Ns, no significant difference, **P*<0.05, ***P*<0.01.

Supplementary Figure S3. TCGA-based correlation analysis between *TMEM9* and *VEGF*.

Supplementary Figure S4. Inhibition of *TMEM9* reduced the expression and secretion of *VEGF*. A QRT–PCR and Western blot analysis of *VEGF* in LUAD stable cell lines overexpressing *TMEM9* (*TMEM9*) or their control cell lines (Vector). **B, C** Western blot and qRT-PCR analysis of *VEGF* in LUAD stable cell lines with

TMEM9 inhibition (sh*TMEM9*) or their control cell lines (sh-NC). Data are presented as the mean ± SD . *P<0.05, **P<0.01.

Supplementary Figure S5. Recombinant *VEGF* (r*VEGF*) abolished the inhibitory effect of *TMEM9*-knockdown LUAD cells on HUVEC angiogenesis and tumor cell migration. A, C, D The EdU assay (A), tube formation (C), and scratch surface healing (D) were examined. r*VEGF* was added to the culture medium of HUVECs coculture with *TMEM9*-knockdown LUAD cells and control cells. **B** The EdU assay was examined. anti*VEGF* was added to the culture medium of HUVECs coculture with *TMEM9* overexpressed LUAD cells and control cells. **E** Effect of r*VEGF* on LUAD cell migration. Results are shown for three experiments performed in triplicate. Data are presented as the mean ± SD. Ns, no significant difference, *P<0.05, **P<0.01.

Supplementary Figure S6. No significant difference of the angiogenesis capacity of lung metastases between *TMEM9* knockdown group and control group. LUAD cells were injected into the tail vein of nude mice (n=5). **A, B** Knockdown of *TMEM9* did not significantly affected microvessel density in lung metastases. **C, D** No significant difference in *VEGF* expression was detected in lung metastases between *TMEM9* knockdown group and control group. Scale bar, 100 μm. Ns, no significant difference.

Corresponding Author Name: Xuezhen Ma
Manuscript Number: ͻIS-23-3067RRR

Reporting Summary

Springer Nature wishes to improve the reproducibility of the work that we publish. This checklist is used to ensure good reporting standards and to improve the reproducibility. Please respond completely to all questions relevant to your manuscript. For more information, please read the journal's Guide to Authors.

☑ **Check here to confirm that the following information is available in the Material & Methods section:**

- **The exact sample size (*n*)** for each experimental group/condition, given as a number, not a range
- **A description of the sample collection** allowing the reader to understand whether the samples represent technical or biological replicates (including how many animals, litters, culture, etc.)
- **A statement of how many times the experiment shown was replicated in the laboratory**
- **Definitions of statistical methods and measures**: For small sample sizes (n<5) descriptive statistics are not appropriate, instead plot individual data points
    - Very common tests, such as *t*-test, simple χ^2 tests, Wilcoxon and Mann-Whitney tests, can be unambiguously identified by name only, but more complex techniques should be described in the methods section
    - Are tests one-sided or two-sided?
    - Are there adjustments for multiple comparisons?
    - **Statistical test results**, e.g., ***P* values**
    - Definition of **'center values'** as **median or mean**;
    - Definition of **error bars as s.d. or s.e.m. or c.i.**

Please ensure that the answers to the following questions are reported in the manuscript itself. We encourage you to include a specific subsection in the methods section for statistics, reagents and animal models. Below, provide the page number or section and paragraph number.

Statistics and general methods

	**Reported in section/paragraph or page #**
1. How was the sample size chosen to ensure adequate power to detect a pre-specified effect size? (Give section/paragraph or page #)	Materials and methods,page 10
For animal studies, include a statement about sample size estimate even if no statistical methods were used.	N/A
2. Describe inclusion/exclusion criteria if samples or animals were excluded from the analysis. Were the criteria pre-established? (Give section/paragraph or page #)	Materials and methods,page 5-6.
3. If a method of randomization was used to determine how samples/animals were allocated to experimental groups and processed, describe it. (Give section/paragraph or page #)	All Mice were randomly assigned to each group in this stud y. Materials and methods,page 5-6.
For animal studies, include a statement about randomization even if no randomization was used.	N/A

4. If the investigator was blinded to the group allocation during the experiment and/or when assessing the outcome, state the extent of blinding. (Give section/paragraph or page #)	N/A
For animal studies, include a statement about blinding even if no blinding was done.	N/A
5. For every figure, are statistical tests justified as appropriate?	Yes, Materials and methods,page 10.
Do the data meet the assumptions of the tests (e.g., normal distribution)?	Yes, Materials and methods,page 10.
Is there an estimate of variation within each group of data?	Yes, the variation has been marked in each figure.
Is the variance similar between the groups that are being statistically compared? (Give section/paragraph or page #)	Yes, Materials and methods,page 10.

Reagents

Reported in section/paragraph or page #

6. Report the source of antibodies (vendor and catalog number)	Materials and methods,page 7-8.
7. Identify the source of cell lines and report if they were recently authenticated (e.g., by STR profiling) and tested for mycoplasma contamination	Materials and methods,page 3.

Animal Models

Reported in section/paragraph or page #

8. Report species, strain, sex and age of animals	Materials and methods,page 5-6.
9. For experiments involving live vertebrates, include a statement of compliance with ethical regulations and identify the committee(s) approving the experiments.	page 6

10. We recommend consulting the ARRIVE guidelines (*PLoS Biol.* **8**(6), e1000412,2010) to ensure that other relevant aspects of animal studies are adequately reported.

Human subjects

Reported in section/paragraph or page #

11. Identify the committee(s) approving the study protocol.	Materials and methods,page 4.
12. Include a statement confirming that informed consent was obtained from all subjects.	Materials and methods,page 4.
13. For publication of patient photos, include a statement confirming that consent to publish was obtained.	N/A
14. Report the clinical trial registration number (at ClinicalTrials.gov or equivalent).	N/A

15. For phase II and III randomized controlled trials, please refer to the CONSORT statement and submit the CONSORT checklist with your submission.

16. For tumor marker prognostic studies, we recommend that you follow the REMARK reporting guidelines.

Data deposition

Reported in section/paragraph or page #

17. Provide accession codes for deposited data. Data deposition in a public repository is mandatory for:<br>a. Protein, DNA and RNA sequences<br>b. Macromolecular structures<br>c. Crystallographic data for small molecules<br>d. Microarray data	N/A

Deposition is strongly recommended for many other datasets for which structured public repositories exist; more details on our data policy are available in the Guide to Authors. We encourage the provision of other source data in supplementary information or in unstructured repositories such as Figshare and Dryad. We encourage publication of Data Descriptors (see Scientific Data) to maximize data reuse.

18. If computer code was used to generate results that are central to the paper's conclusions, include a statement in the Methods section under "**Code availability**" to indicate whether and how the code can be accessed. Include version information as necessary and any restrictions on availability.	N/A

Table S1 Comparison of pathological data between the two groups of patients with LUAD

Characteristics	Total patients(n,%)	*TMEM9* expression		
		Low（n=20）	High（n=23）	*P* value
**Gender**				0.818
Male	18(41.9%)	8	10	
Female	25(58.1%)	12	13	
**Age,year**				0.954
＜60	17(39.5%)	8	9	
≥60	26(60.5%)	12	14	
**T stage**				**0.029**
T1	30(69.8%)	18	12	
T2	8(18.6%)	1	7	
T3	4(9.3%)	1	3	
T4	1(2.3%)	0	1	
**N stage**				0.111
N0	37(86%)	17	20	
N1	2(4.7%)	1	1	
N2	4 (9.3%)	2	2	
**M stage**				
M0	43(100%)	20	23	
M1	0(0%)	0	0	
**Clinical stage**				0.147
I	35(81.4%)	15	20	
II	4(9.3%)	2	2	
III	4(9.3%)	3	1	
IV	0 (0%)	0	0	
**STAS**				**0.001**
Negative	28(65.1%)	18	10	
Positive	15(34.9%)	2	13	
**Cigarette smoking history, pack year**				0.59
0	27(62.8%)	11	16	
＜10	4(9.3%)	2	2	
≥10	12(27.9%)	7	5	

TMEM9, transmembrane protein 9; LUAD, lung adenocarcinoma; STAS, spread through air spaces.

Table S2 Comparison of pathological data between the two groups of patients with LUAD (HLugA180Su11)

Characteristics	Total patients(n,%)	*TMEM9* expression		
		Low（n=45）	High（n=45）	*P* value
**Gender**				1.000
Male	48（53.3%）	24	24	
Female	42（46.7%）	21	21	
**Age,year**				0.203
＜60	40（44.4%）	23	17	
≥60	50（55.6%）	22	28	
**T stage**				**0.006**
T1	51 (56.7%)	33	18	
T2	29 (32.2%)	9	20	
T3-4	10 (11.1%)	3	7	
**N stage**				0.192
Negative	56（62.2%）	31	25	
Positive	34（37.8%）	14	20	
**M stage**				
M0	90（100%）	45	45	
M1	0（0%）	0	0	
**Clinical stage**				**0.043**
I -II	70 (77.8%)	39	31	
III-Ⅳ	20 (22.2%)	6	14	
**Histologic grades**				**0.006**
1-2	69 (76.7%)	40	29	
3	21 (23.3%)	5	16	

TMEM9, transmembrane protein 9; LUAD, lung adenocarcinoma.

Table S3 Clinical characteristics of patients (HLugA120PG01)

Characteristics	Total patients(n,%)
**Gender**	
Male	47（39.2%）
Female	73（60.8%）
**Age,year**	
＜60	41（34.2%）
≥60	79（65.8%）
**T stage**	
T1	97 (80.8%)
T2	11 (9.2%)
T3-4	7 (5.8%)
unknown	5(4.2%)
**Histologic grades**	
1	40(33.3%)
2	67 (55.8%)
3	13 (10.9%)
