# Supplementary figures and images for: TMEM9 promotes lung adenocarcinoma progression via activating the MEK/ERK/STAT3 pathway to induce VEGF expression
Source: Cell Death Dis. 2024 Apr 25;15(4):295. doi: 10.1038/s41419-024-06669-8 (PMC11045738; doi:10.1038/s41419-024-06669-8)

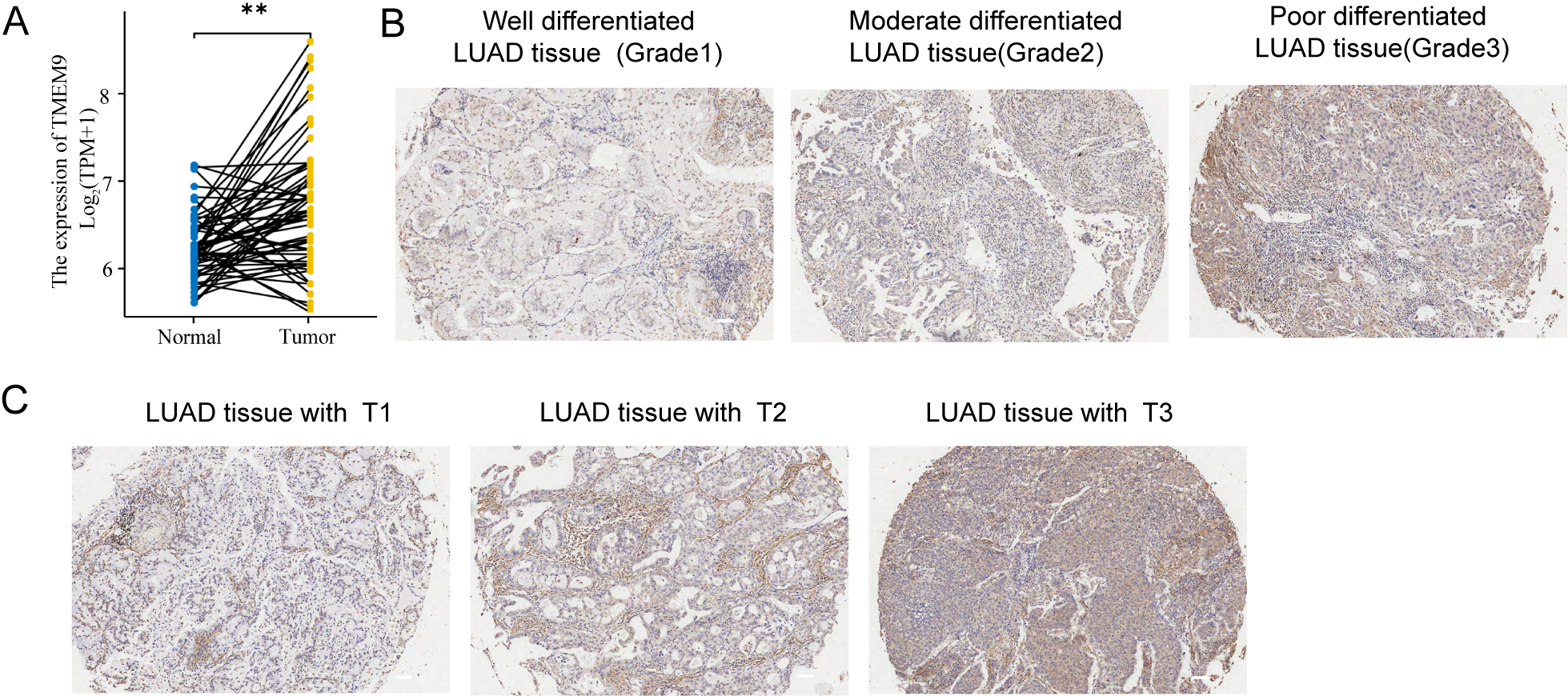

Supplement: Supplementary file 2 — Supplementary Figure S1 [file 41419_2024_6669_MOESM2_ESM.tif]

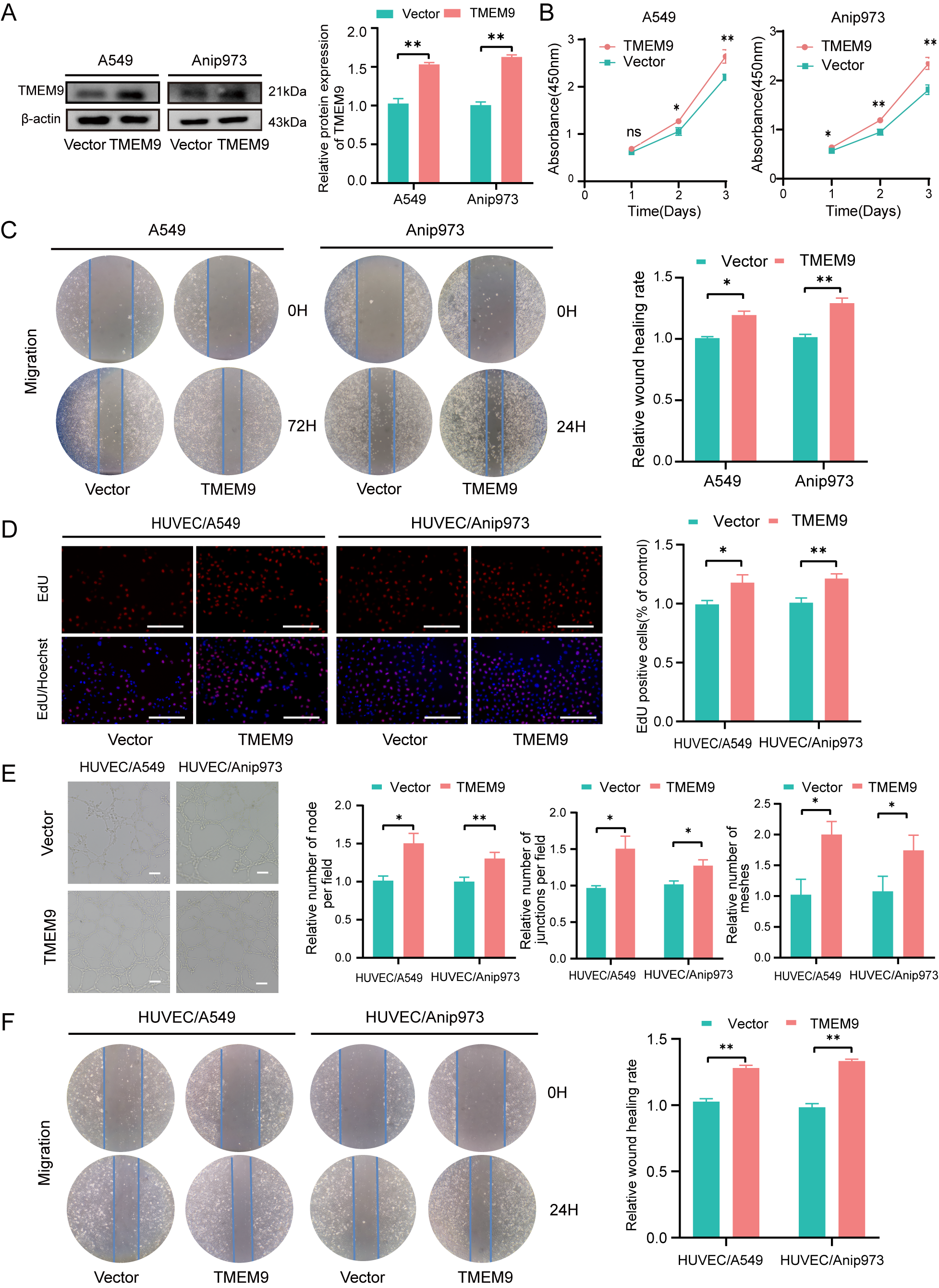

Supplement: Supplementary file 3 — Supplementary Figure S2 [file 41419_2024_6669_MOESM3_ESM.tif]

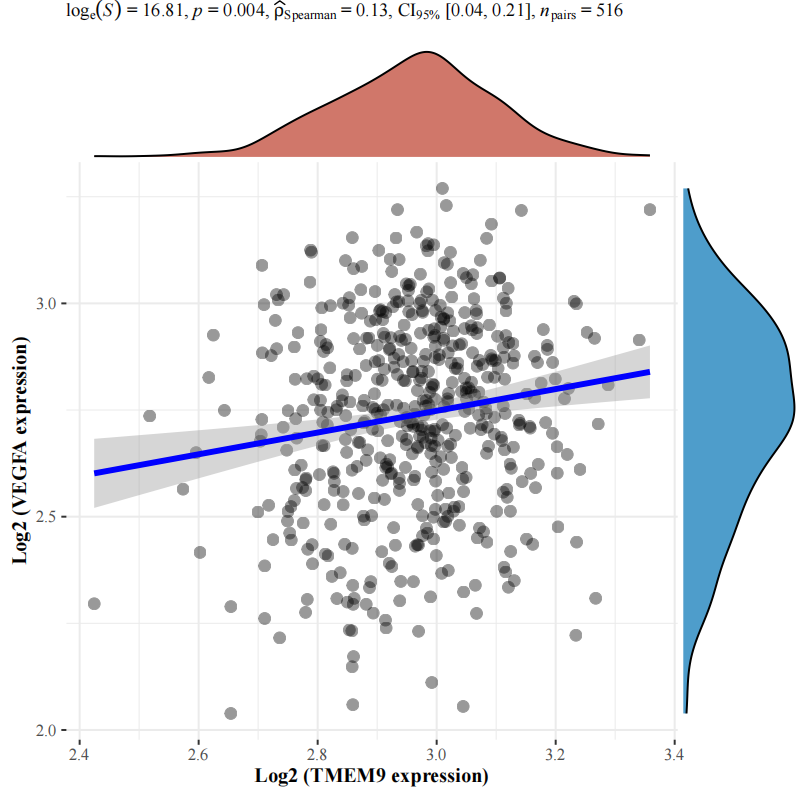

Supplement: Supplementary file 4 — Supplementary Figure S3 [file 41419_2024_6669_MOESM4_ESM.tif]

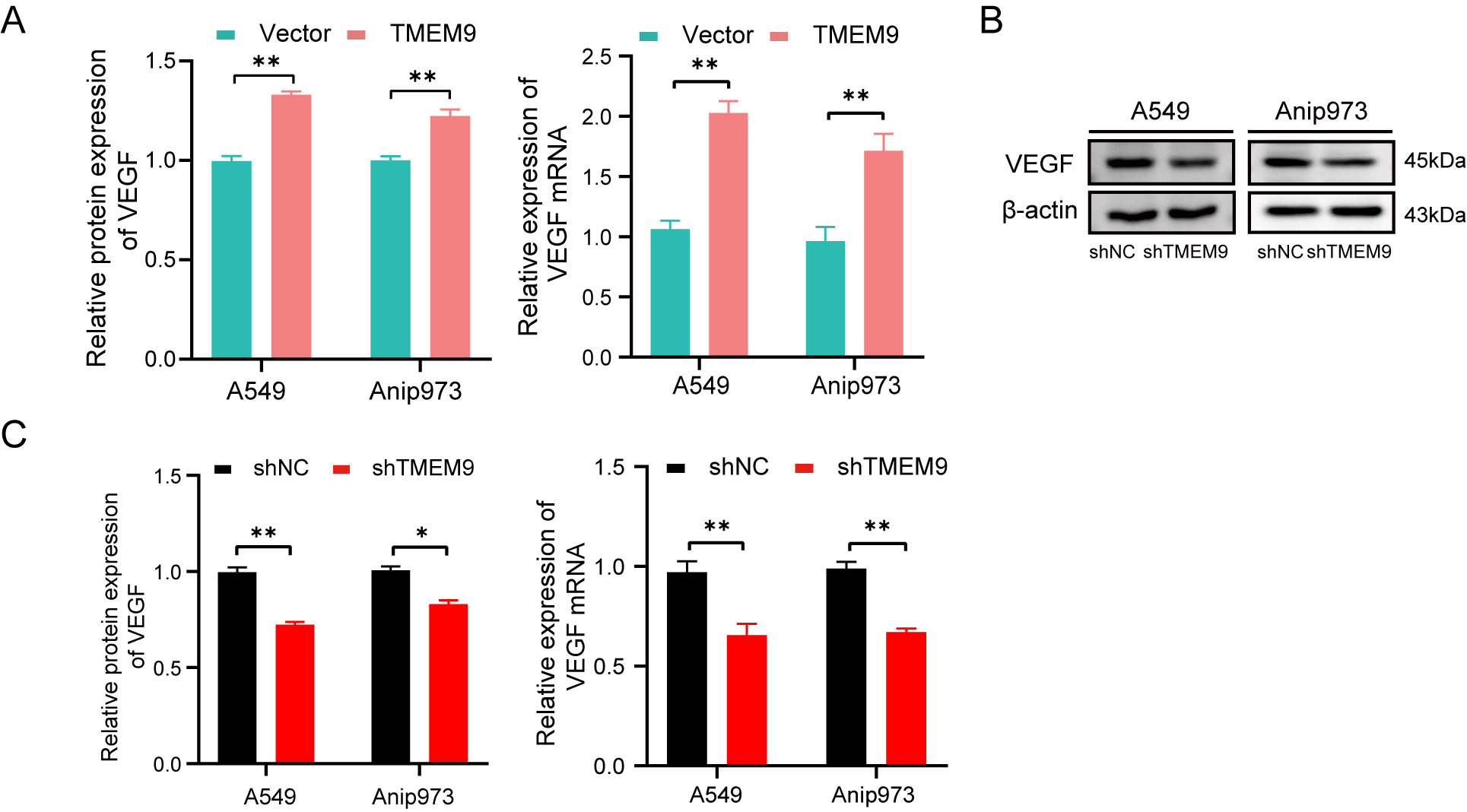

Supplement: Supplementary file 5 — Supplementary Figure S4 [file 41419_2024_6669_MOESM5_ESM.tif]

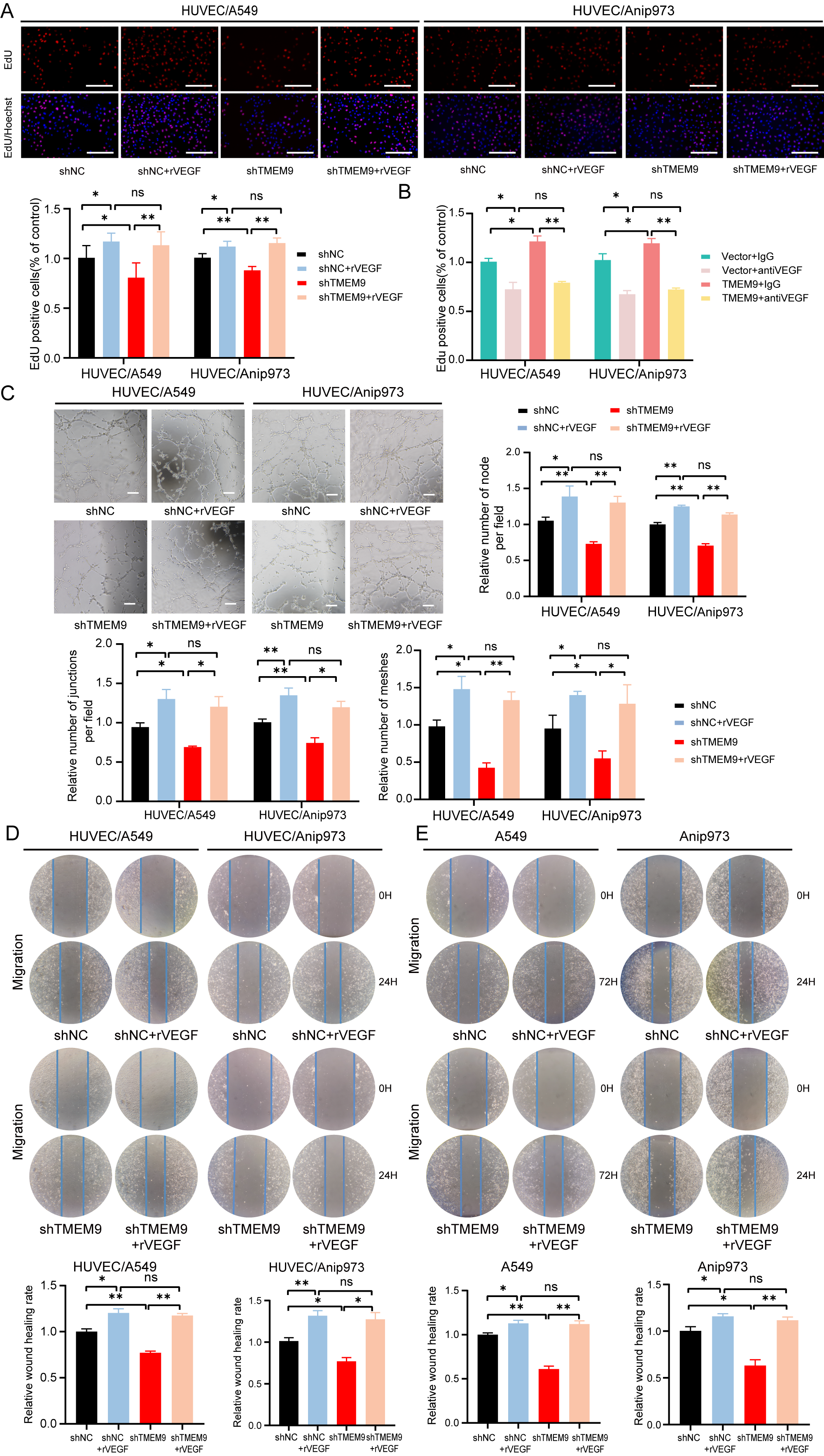

Supplement: Supplementary file 6 — Supplementary Figure S5 [file 41419_2024_6669_MOESM6_ESM.tif]

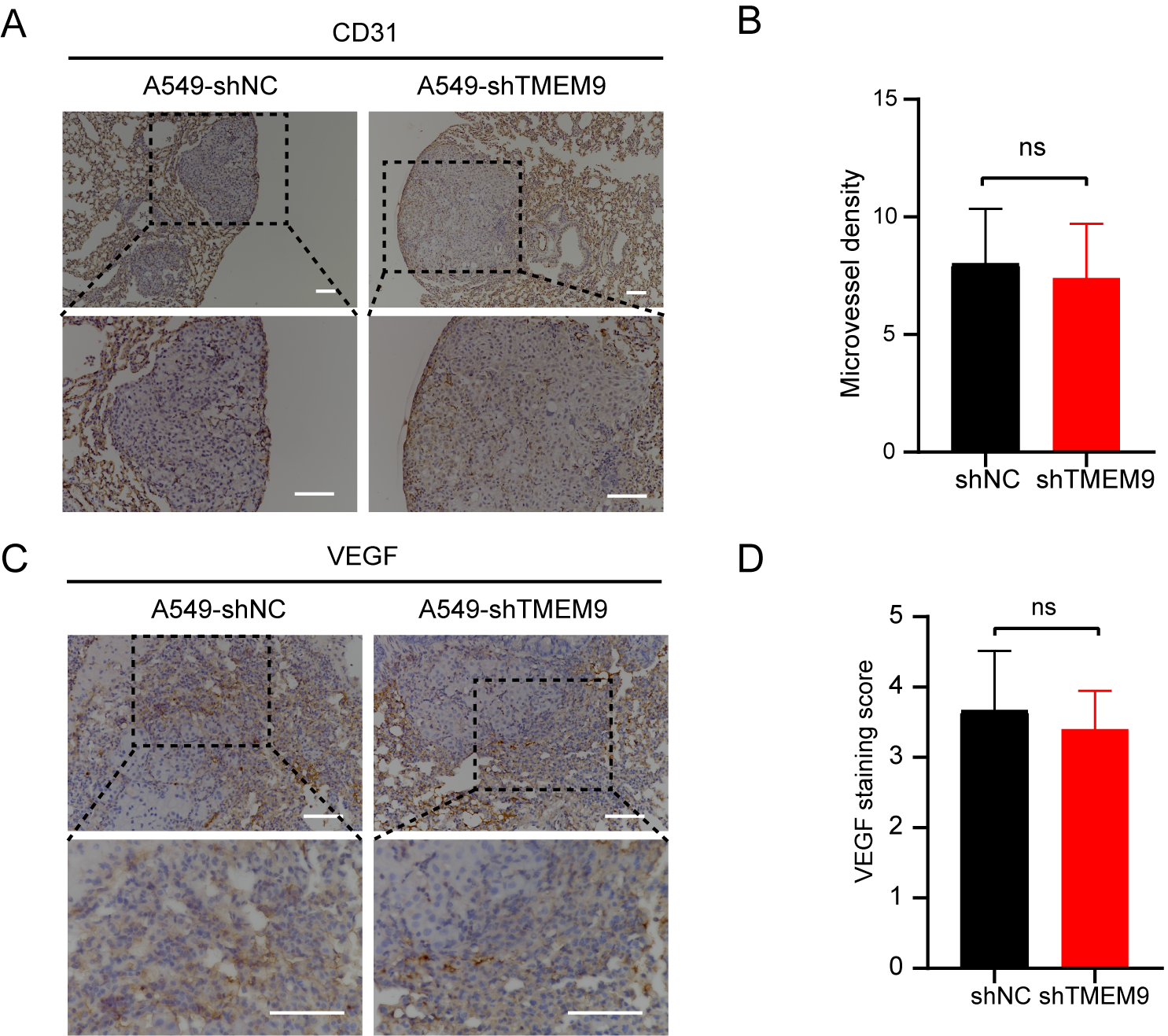

Supplement: Supplementary file 7 — Supplementary Figure S6 [file 41419_2024_6669_MOESM7_ESM.tif]
